# Allele-Independent Turnover of Human Leukocyte Antigen (HLA) Class Ia Molecules

**DOI:** 10.1371/journal.pone.0161011

**Published:** 2016-08-16

**Authors:** Claudia Prevosto, M. Farooq Usmani, Sarah McDonald, Aleksandra M. Gumienny, Tim Key, Reyna S. Goodman, J. S. Hill Gaston, Michael J. Deery, Robert Busch

**Affiliations:** 1 Department of Medicine, University of Cambridge, Cambridge, United Kingdom; 2 Tissue Typing Laboratory, Addenbrooke’s Hospital, Cambridge, United Kingdom; 3 Cambridge Centre for Proteomics, University of Cambridge, Cambridge, United Kingdom; 4 Department of Life Sciences, University of Roehampton, London, United Kingdom; Hospital Israelita Albert Einstein, BRAZIL

## Abstract

Major histocompatibility complex class I (MHCI) glycoproteins present cytosolic peptides to CD8+ T cells and regulate NK cell activity. Their heavy chains (HC) are expressed from up to three MHC gene loci (human leukocyte antigen [HLA]-A, -B, and -C in humans), whose extensive polymorphism maps predominantly to the antigen-binding groove, diversifying the bound peptide repertoire. Codominant expression of MHCI alleles is thus functionally critical, but how it is regulated is not fully understood. Here, we have examined the effect of polymorphism on the turnover rates of MHCI molecules in cell lines with functional MHCI peptide loading pathways and in monocyte-derived dendritic cells (MoDCs). Proteins were labeled biosynthetically with heavy water (^2^H_2_O), folded MHCI molecules immunoprecipitated, and tryptic digests analysed by mass spectrometry. MHCI-derived peptides were assigned to specific alleles and isotypes, and turnover rates quantified by ^2^H incorporation, after correcting for cell growth. MHCI turnover half-lives ranged from undetectable to a few hours, depending on cell type, activation state, donor, and MHCI isotype. However, in all settings, the turnover half-lives of alleles of the same isotype were similar. Thus, MHCI protein turnover rates appear to be allele-independent in normal human cells. We propose that this is an important feature enabling the normal function and codominant expression of MHCI alleles.

## Introduction

Classical MHC class Ia (MHCI) membrane glycoproteins are expressed on most nucleated cells [1, 2]. Their maturation begins with the assembly of its constituent heavy chain (HC) with the β_2_-microglobulin (β_2_m) light chain in the endoplasmic reticulum (ER) [3]. MHCI heterodimers associate with a peptide loading complex, comprising the transporter associated with antigen presentation (TAP), tapasin, and chaperones [3]. TAP imports diverse peptides, derived from cytosolic protein turnover, into the ER [4, 5], which may be further trimmed by ER aminopeptidases (ERAP1 and 2 in humans) before occupying the peptide-binding groove of MHCI molecules [6], and edited through peptide exchange catalysed by TAPBPR or tapasin [7, 8]. Bound peptides are restricted in length and exhibit sequence preferences, generally at the second and penultimate position, reflecting interactions with specificity pockets in the groove [9]. Peptide-loaded MHCI molecules exit the ER and are expressed at the cell surface. The main components of this pathway are conserved between humans and rodents [5].

Thus, MHCI proteins present cytosolic peptides for recognition by cognate αβ T-cell antigen receptors (TCRs) of CD8+ T cells, enabling adaptive immune surveillance of cytosolic pathogens, as well as establishment of tolerance to self peptides in the CD8+ T-cell repertoire [1, 10]. In addition, both classical and non-classical MHC class I molecules engage NK cell receptors of the lectin and Ig superfamilies [11]. Normally, inhibitory receptor interactions predominate, allowing normal tissue cells to avoid NK cell-mediated killing; loss of MHCI surface expression in stressed or virally infected cells releases this inhibition [12].

Classical MHCI HC genes are remarkably polymorphic, with thousands of alleles at each of three loci (HLA-A, -B, -C in humans) expressed in the population (http://www.ebi.ac.uk/ipd/imgt/hla/stats.html)[13]. Alleles at any one locus are typically distinguished by multiple amino acid differences, many of which affect specificity pockets in the groove [10, 14, 15]. This polymorphism diversifies the peptides that can be presented to CD8+ T cells and is maintained by pathogen-mediated selection [16]. The functional benefits require codominant expression of the maternally and paternally inherited alleles [1]. Nonetheless, allelic variants at the same MHC class I locus are not necessarily transcribed equally [17, 18], correlating with unequal quantities being expressed at the cell surface [18]. Any such allelic differences are superimposed on the differential expression of the classical MHCI loci, with HLA-A and -B proteins usually being expressed more highly than HLA-C [19–21]. This pattern may be modified in specific cell types, such as activated natural killer (NK) cells, which have recently been shown to downregulate HLA-A mRNA selectively [22].

Given that protein levels are determined by the balance of production and destruction, an important related question is whether MHCI alleles and loci differ in their rates of degradation. This is plausible because closely related MHCI alleles differ in their thermal stability, conformational dynamics, interactions with the peptide loading complex, and rates of assembly and egress from the ER [23–27]. Consequently, MHCI alleles also differ in their turnover rates when expressed in mutant cell lines with peptide loading defects. Viral immune evasion mechanisms that target MHCI degradation may also act in an allele-specific manner, either because they interfere with the peptide-loading complex (which, as explained above, affects some MHCI alleles more than others) or because viral immunoevasins interact directly with polymorphic segments of the MHCI heavy chain [28, 29]. Locus differences in the fate of MHCI molecules are suggested by studies in transfectants, showing that the HLA-C cytoplasmic tail mediates faster rates of endocytosis and lysosomal degradation than the cytoplasmic tails of HLA-A or -B [30]. Turnover can also be modulated by viral infection in an isotype-specific manner [31]. Little is known, however, about the effect of allelic or isotypic diversity on MHCI protein degradation in unmanipulated cells with intact peptide loading.

Several disposal pathways for MHCI molecules have been identified. Misfolded MHCI HCs are retained in the ER and degraded by ER-associated degradation (ERAD) [32, 33]. In contrast, folded MHCI HC/β_2_m complexes are degraded post-Golgi, by internalization from the plasma membrane, followed by lysosomal degradation [30, 34]. Viral immune evasion mechanisms may target MHCI molecules to either pathway [29]. However, competing with degradation, some internalized MHCI molecules may undergo peptide exchange and return to the plasma membrane [35]. Shedding into the extracellular space is a third mechanism of loss from the cell surface [36].

The effect of polymorphism on MHCI turnover is difficult to measure by ^35^S-pulse/chase radiolabeling, a standard approach for measuring protein half-lives [37]. In addition to general limitations of this technique, the best-characterized immunoprecipitating monoclonal antibodies (mAbs) do not discriminate between MHCI alleles and isotypes, nor are they resolved by SDS-PAGE. On the other hand, the conformational preferences of the available allele-specific mAbs could confound the analysis. Here, we have used **s**table **i**sotope labeling of **n**on**e**ssential amino acids with heavy **w**ater (SINEW), an approach developed by us [38] as well as others [39–42], to assess the effect of structural polymorphism on MHCI protein turnover in human cells. A preliminary analysis of these data has previously been published in thesis form [43].

## Materials and Methods

### Human subjects

All studies involving human subjects were performed in accordance with the Declaration of Helsinki, with approval of the Cambridge Regional Ethical Committee (01/363). All donors gave written informed consent. Healthy donors (HD, n = 9, of whom 5 had previously been identified as HLA-B27 positive) and one B27-negative patient with ankylosing spondylitis were recruited. The cohort was enriched for B27+ donors in view of recent research linking the post-translational fate of this allele to the pathogenesis of spondyloarthropathy (cf. Discussion).

In one instance, leukocytes were obtained from the buffy coat of a healthy blood donor following plasmapheresis (NHS Blood and Transplant, UK). From the other donors, peripheral blood (50 ml) was obtained by venepuncture and mixed with sterile sodium heparin (2 U/ml final concentration; Wockhardt, Wrexham, UK) as anticoagulant.

### Cell culture and ^2^H_2_O labeling

The acute myeloid leukemia cell line, KG-1 [44], was grown in IMDM containing 20% heat-inactivated fetal bovine serum (FBS), 2 mM glutamine, and antibiotics in a humidified incubator at 5% CO_2_ and 37°C. The Epstein-Barr virus (EBV)-transformed B-cell line, LCL721 [45], was grown in RPMI1640 with 10% FBS, 2 mM glutamine and antibiotics. Cells were stored in liquid nitrogen using FBS with 10% v/v DMSO as cryopreservative. Both cell lines grew in suspension and were maintained in exponential growth by diluting in warm media every 2–4 days. Viability was routinely > 95%.

^2^H_2_O labeling [38] was initiated by mixing one volume of cell suspension with one volume of complete growth media containing 10% v/v ^2^H_2_O (99% atom per cent enrichment (a.p.e.); final enrichment ≈ 5%; from Cambridge Isotope Labs, Tewksbury, MA, USA). ^2^H_2_O enrichment was subsequently maintained, usually for at least five doublings, by further dilution with media containing 5% v/v ^2^H_2_O, at a rate approximating the cell growth rate to maintain cells near steady state. For use in cell culture, ^2^H_2_O was adjusted to 0.9% w/v NaCl, sterile filtered, and LPS depleted by passage over polymyxin B columns (DetoxiGel, Thermo Scientific, Paisley, UK) before addition to media. Samples were removed, counted by hemocytometer or a hand-held cell counting device (Scepter 2.0, Millipore, Watford, UK), washed in ice-cold PBS, and stored at or below -20°C for protein analysis. For inhibition of lysosomal degradation, KG-1 cells were cultured in the presence of 25 mM ammonium chloride for up to 3 days.

MoDC cultures were prepared as previously described [46, 47]. Briefly, peripheral blood mononuclear cells (PBMCs) were obtained by Ficoll density gradient centrifugation of whole blood or buffy coat cells according to the manufacturer’s instructions (GE Life Sciences, Little Chalfont, UK), harvested from the gradient interface, and washed in low-endotoxin PBS. CD14+ monocytes were enriched by positive selection with anti-CD14-coated MACS microbeads and a MACS separator, as per manufacturer’s instructions (Miltenyi Biotec, Bisley, UK). Monocytes were cultured at 0.25 × 10^6^ cells/ml in 25 cm^2^ tissue culture dishes (0.5–1.5 × 10^6^ cells per time point) in RPMI 1640 with 10% fetal bovine serum, 2 mM glutamine, and antibiotics, and differentiated with 50 ng/ml recombinant human GM-CSF and 1000 U/ml recombinant human IL-4 for 6 days. The resultant MoDCs were stimulated with or without 100 ng/ml *Escherichia coli* 026:B6 lipopolysaccharide (LPS; cat no. L2654, Sigma Aldrich, Dorset, UK). ^2^H_2_O labeling was initiated 24 hours later and continued for up to 72 hours. DC-like morphology was confirmed by visual inspection. Supernatant and loosely adherent cells were harvested, and more tightly adherent cells were harvested by brief incubation in PBS with trypsin/EDTA at 37°C. The cells were pooled, washed in PBS, and used for flow cytometric and biochemical analysis.

### Flow cytometry

Monocyte purity and DC differentiation from monocytes were verified by surface staining, confirming loss of CD14, gain of CD11c, and expression of CD11b and CD1a (not shown). Following the differentiation step, MoDC activation was confirmed by surface staining for HLA-DR (L243-FITC) and CD86 (PE). In all experiments, expression of folded HLA-A/-B/-C molecules was monitored by staining with saturating concentrations of W6/32 mAb (protein A-purified from hybridoma supernatant, and PE-conjugated) [48] in PBS/1% BSA/0.05% sodium azide. Cells were incubated with fluorescent mAb conjugates for 30 minutes at 4°C, followed by washing. KG-1 cells were analysed either live or after fixation and permeabilisation (Cytofix/Cytoperm, BD Europe, Oxford, UK) and after blocking of F_c_ receptors with 10% normal mouse serum. Samples were analysed on a FACSCantoII flow cytometer running FACSDiVa software (BD Europe). Data were exported to FlowJo (FlowJo, Ashland, OR, USA) for color compensation and data analysis. Percentages and median fluorescence intensities were determined after gating on intact cells by forward and side scatter, with doublet exclusion by gating on forward scatter height vs. area.

### MHC protein isolation

This was done as previously described [38]. Briefly, ^2^H_2_O-labeled cell pellets were extracted in ice-cold TBS, pH 8.0, containing 1% CHAPS, Roche (Burgess Hill, UK) Complete protease inhibitors, and 10 mM iodoacetamide, and incubated for 1 hour at 4°C, mixing continuously. Extracts contained 10–25 × 10^6^ immortalized cell equivalents per ml, or 0.5–1.5 × 10^6^ MoDCs per 0.5 ml, per time point. Insoluble material was spun out. Extracts were precleared with protein A-sepharose before immunoprecipitation with 10–30 μg W6/32 mAb and protein A-sepharose. In addition, HLA-DR was immunoprecipitated from MoDC extracts, using the L243 mAb. Immunoprecipitates were extensively washed in 0.1% CHAPS buffer, boiled in nonreducing SDS-PAGE sample buffer, and resolved on 12% acrylamide SDS-PAGE gels. Proteins were visualized by Instant Blue^™^ (Expedeon, Swavesey, UK) or silver staining. MHCI HC and DR α chain bands were identified by molecular weight and comparison to IgG2a isotype control immunoprecipitations, excised, and stored at -80°C.

### Measurement of ^2^H_2_O enrichment in media

Sera and media were diluted gravimetrically. Their ^2^H_2_O enrichment was quantified by isotope ratio mass spectrometry at the Mass Spectrometry Laboratory of the Medical Research Council Human Nutrition Laboratories (Fulbourn, UK), as previously described [38], and averaged across all samples from each labeling time course.

### Liquid chromatography/tandem mass spectrometry (LC-MS/MS)

In-gel reduction, derivatization of cysteines with iodoacetamide, and tryptic digestion of bands of interest were performed at the Cambridge Centre for Proteomics, as previously described [38]. LC-MS/MS analysis of digests was performed using a LTQ Orbitrap Velos tandem mass spectrometer (Thermo Scientific, San Jose, CA)[49]. Data were acquired in data-dependent acquisition (DDA) mode. Mass spectra of intact peptide ions (2+ to 4+ charge) were acquired in the Orbitrap detector set to 7500 resolution (defined at mass to charge [*m/z*] = 400). For peptide identification, tandem MS (LC-MS/MS) data were acquired after collision-induced dissociation of the five most abundant peptides in each MS scan. MHC protein-derived peptides were identified using the Mascot (Matrix Science, London, UK) search algorithm, with a mass tolerance of 25 ppm, carboxamidomethylation of cysteines as a fixed modification, and methionine oxidation as a variable modification.

### HLA typing and assignment of peptides to MHCI alleles and isotypes

Cell lines and whole blood or CD14-depleted PBMCs from donors were genotyped for HLA-A, -B, and -C using PCR-SSP at the Tissue Typing Laboratory, Addenbrooke’s Hospital. In this paper, asterisks and leading zeros used in HLA nomenclature are generally omitted when discussing protein variants, but included where genotypes are specifically referenced. Where genotypes and the corresponding serotypes differ in numbering, both designations are given in the **Supporting Information** (S3 Table) and text.

In each case, all peptide sequences detected by the Mascot search were explained by the HLA genotype (not shown). The Mascot search algorithm was not, however, sufficient for the task of peptide assignment: it generated false-positive matches to other MHCI alleles, which were not present in the HLA genotype of the cells being analysed. This was due to matches of the identified peptides, in novel combinations, to irrelevant MHCI alleles in the sequence databases. Moreover, some peptides were shared by more than one isotype amongst the alleles that were present.

In order to address these issues, the cDNA sequences of the relevant HLA-A, -B, and -C alleles for each donor were obtained from the IMGT/HLA database (http://www.ebi.ac.uk/ipd/imgt/hla/)[13], and subjected to *in silico* tryptic digestion with Peptide_Mass (http://web.expasy.org/peptide_mass/)[50]. Incomplete cleavages were allowed. Virtual digests were used to identify the subset of peptides that, in any one donor or cell line, are specific for a particular HLA class I allele, or shared by the two alleles of one isotype (but absent from the other two isotypes). Such peptides were then selected from amongst the observed MHCI HC-derived peptides and a selection used for analysis of ^2^H labelling, as recorded in S1–S3 Tables.

### Quantification of fractional protein synthesis

At baseline, peptides exhibit mass heterogeneity due to the random incorporation of stable isotopes at their natural abundance, with the greatest contribution from ^13^C (≈ 1.09% in natural carbon). ^2^H label incorporation into peptides of interest from ^2^H_2_O causes predictable changes in the distribution of mass variants (mass isotopomers) from their baseline values [38]. To quantify these changes, mass isotopomer distributions were determined by integration of peptide ion chromatograms for the monoisotopic base peak (m_0_) and higher mass isotopomers (m_1_, m_2_, …). Fractional molar abundances were calculated by normalization to the total abundance in the mass envelope, and compared to theoretical values, which were calculated by mass isotopomer distribution analysis (MIDA [51]), as implemented in Microsoft Excel (R.B., unpublished; available on request). The goodness of fit between observed and theoretical mass isotopomer distributions was assessed using root-mean-square deviations (RMSD). Mass isotopomer distributions progressively deviated from the unlabeled baseline during ^2^H_2_O labeling, approaching a plateau after extensive labeling. The maximal (plateau) shifts from the baseline were modeled by MIDA, using the measured ^2^H_2_O enrichment in media to represent the precursor pool enrichment, and adjusting the number of biosynthetic ^2^H incorporation sites (n) to minimize the root-mean-square deviation (RMSD) between model and data [38]. Fractional protein synthesis (*f*) was calculated as the percentage of the maximal possible shift of each mass isotopomer from its baseline fractional abundance. Due to the close similarity between theoretical and measured baseline and fully-labeled mass isotopomer distributions, either could be used equivalently for calculation of *f*. Mass isotopomers with an abundance difference below ≈ 8% between the unlabeled and fully-labeled distribution gave poor estimates of *f* and were excluded from calculations. Analytical error for each peptide was estimated as the SD of all informative mass isotopomers (usually 2–4). The data were further inspected to exclude peptides liable to yield poor estimates of *f*, due to low total ion abundance, significant peak contamination, unacceptable levels of divergence from MIDA models (high RMSD), or high SD [49].

### Calculation of turnover rates and statistical analysis

Statistical analysis was performed using Prism software (version 4.0; GraphPad, La Jolla, CA, USA). Fractional synthesis rates (*k*_obs_) were determined by modelling the time course of fractional protein synthesis as a single-exponential rise to maximum,

$$f\left(t\right)=1-e^{-kobs\times t},$$

where *k*_*obs*_ = ln(2)/t_1/2_ and t_1/2_ represents the time required for 50% replacement or dilution of old by new molecules. Best-fit values of *k*_*obs*_ were determined by nonlinear least-squares fit to the time course of *f*. Unless indicated otherwise, analysis of different peptides tracking the same MHCI allele or isotype yielded statistically indistinguishable estimates of *k*_*obs*_ (p > 0.05 by F test), and pooled data were used to calculate fractional synthesis rates and their associated standard errors (SEM) and 95% confidence intervals. Nominally significant differences (p < 0.05) were reported without correction for multiple comparisons.

The fractional synthesis rate equals the protein turnover rate in non-proliferating cells, such as MoDCs, as long as protein levels per cell remain at steady state. For immortalized, exponentially growing cell lines with protein levels at steady state, the contribution of cell growth to fractional protein synthesis was calculated as:

$$f\text{cell}\left(t\right)=1-e^{-kcell\;\times \;t},$$

where *f*_*cell*_ is the proportion of new cells accrued at time t, *k*_*cell*_ = ln(2)/t_2_ is the corresponding fractional cell growth rate, and t_2_ is the cell doubling time calculated from exponential cell growth curves. Given the excellent viability of the cell lines, cell death was assumed to be negligible. In this setting, the contributions from cell growth and protein turnover to the observed fractional protein synthesis are additive:

$$k_{\text{obs}}\;=\;k_{\text{cell}}\;+\;k_{\text{TO}},$$

where *k*_*TO*_ represents the fractional protein turnover rate, related to the turnover half-life as *k*_*TO*_ = ln(2)/t_1/2TO_.

## Results

### Experimental approach

**Fig 1:**
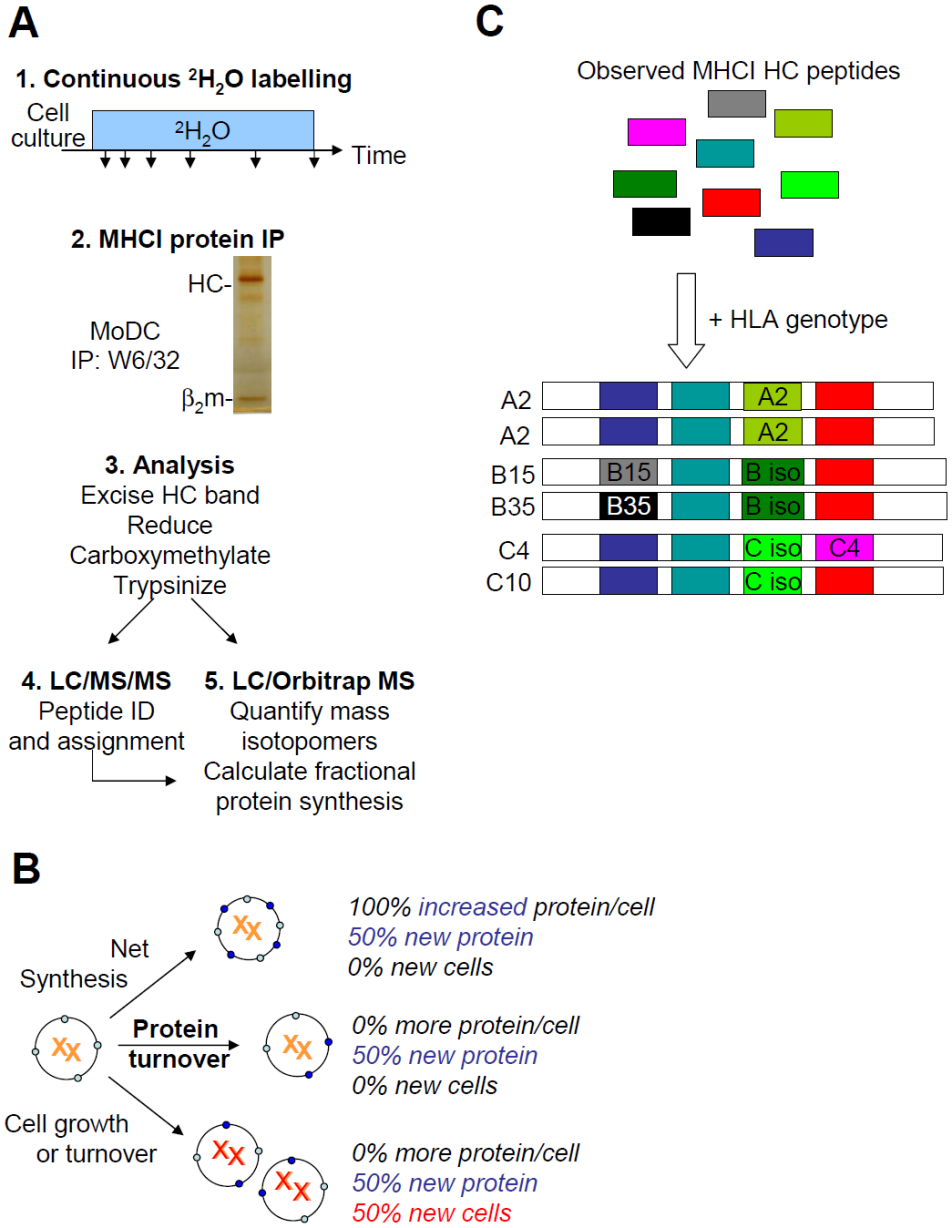
Measuring MHCI protein turnover by ^2^H_2_O labeling. (A) SINEW work flow. See text for details. (B) New protein synthesis may support cell growth, increase net protein levels per cell, or replace protein lost to turnover. These processes contribute additively to protein synthesis. (C) Assignment of peptides to particular MHCI alleles or isotypes. First, LC-MS/MS data (*Step 4* in panel A) are screened against sequence databases to identify tryptic fragments derived from *any* MHCI molecules (*top*, color-coded boxes). MHCI alleles present in each donor are identified by HLA genotyping, and their predicted amino acid sequences are subjected to tryptic digestion *in silico*. These virtual digests are compared with each other to identify a subset of peptides that are specific to particular alleles or isotypes (symbolised by boxes with text labels).

To quantify MHCI fractional synthesis rates, biosynthetic heavy water labeling (SINEW) was performed [38, 49]. Cells were cultured for varying amounts of time in the presence of ≈ 5% heavy water (^2^H_2_O; Fig 1A, step 1). Folded, β2m-associated HLA-A, -B, and -C molecules were immunoprecipitated with the conformation-dependent mAb, W6/32, and analysed by SDS-PAGE (step 2). The MHCI HC band was excised and processed for mass spectrometric analysis (step 3). Tandem mass spectrometry was used to identify tryptic peptides suitable for analysis (step 4). Their ^2^H incorporation was tracked by LC-MS to quantify fractional protein synthesis (step 5).

In cell lines in steady-state growth, protein synthesis may occur either to replace protein lost to turnover, or to maintain protein expression per cell as the cells divide. Thus, protein turnover in proliferating cells can be determined from the excess of the fractional synthesis rate over the cell growth rate. When protein levels per cell increase over time, the biosynthetic contribution to protein accumulation also must be accounted for. These additive contributions are schematically illustrated in Fig 1B.

### MHCI turnover in KG-1 cells

Using this approach, MHCI protein turnover rates were determined in KG-1 cells, an acute myeloid leukemia cell line, which has been used in studies of MHCI post-translational fate and B27 misfolding [34, 52] and as a model of differentiation into dendritic cell-like phenotypes [53]. Here, proliferating, undifferentiated KG-1 cells were used. LC-MS/MS analysis of MHCI HC tryptic digests identified a number of peptides, all of which were explained by the HLA genotype of this cell line. There was no evidence for contamination by non-classical MHCI (Class Ib) molecules (not shown). Some of the observed MHCI-derived tryptic fragments were allele-specific, whereas others were shared between the two alleles of a single isotype, but not by any other of the MHCI alleles present. Yet other peptides, however, were shared by more than one isotype (schematically illustrated in Fig 1C). The mixed origin of these peptides would be expected to confound the analysis, and accordingly they were not studied further. S1 Table lists a subset of peptides that were either allele- or isotype-specific, and that in addition were suitable for quantification of fractional synthesis rates, according to the analytical criteria described in Materials and Methods.

**Fig 2:**
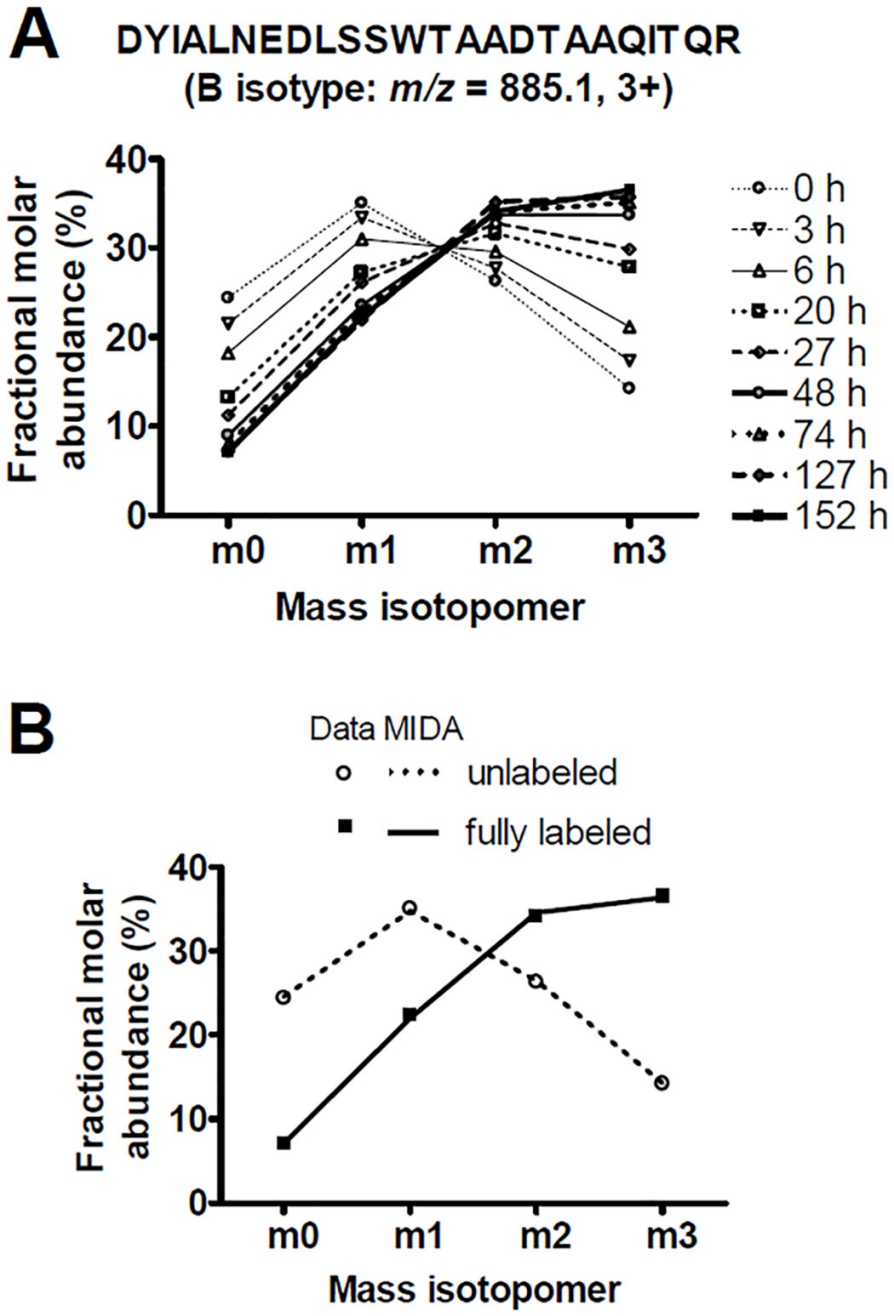
Effect of ^2^H_2_O labeling on peptide mass isotopomer distributions. (A) Mass isotopomer distributions of a MHCI-derived, B isotype-specific tryptic peptide from KG-1 cells after labeling with ^2^H_2_O for various times. Lines connect data at each time point. Within error, each mass isotopomer changed from its initial (unlabeled) to final plateau (fully-labeled) value at the same rate, which is identical to the rate of protein fractional synthesis. (B) For the same peptide, MIDA models for the unlabeled and fully-labeled mass isotopomer distributions (dashed and solid lines, respectively) were compared with experimental data (symbols). RMSD values were 0.20% and 0.25%, respectively, for unlabeled and fully-labeled samples).

Even without isotopic labeling, peptides exhibit mass heterogeneity due to natural isotopes. During ^2^H_2_O labeling of proteins, ^2^H atoms are incorporated into C-H bonds of nonessential amino acids, and hence into newly synthesized proteins, shifting their relative abundances towards the heavier mass variants (“mass isotopomers”), at the expense of the lighter ones. An example is shown in Fig 2A. Each of the mass isotopomers changed its relative abundance over the labeling time course at the same rate (within experimental error). After a few days of labelling, the shift in the distribution approached a plateau, representing complete replacement of old by newly synthesized protein. As described, the intermediate distributions can be modelled as a weighted average of the unlabeled and fully-labeled distributions, weighted in proportion to the fraction of new (labeled) proteins present. Fractional protein synthesis and the associated measurement error can thus be estimated [38].

The relative abundances of mass isotopomers (i.e., the mass isotopomer distribution) can be predicted accurately by MIDA (cf. Materials and Methods [38, 51]). Fig 2B shows that both the unlabeled and fully-labeled distributions observed in the mass spectra of this peptide (*symbols*) were in close agreement with MIDA models (*lines*). The unlabeled MIDA model has no adjustable parameters; its close agreement with experiment (low RMSD) reflects measurement accuracy. The fully-labeled MIDA model has one adjustable parameter, representing an effective number of sites at which ^2^H may be incorporated from ^2^H_2_O. These metrics are reported for each peptide in S1 Table, along with the total ion abundance of each peptide.

**Fig 3:**
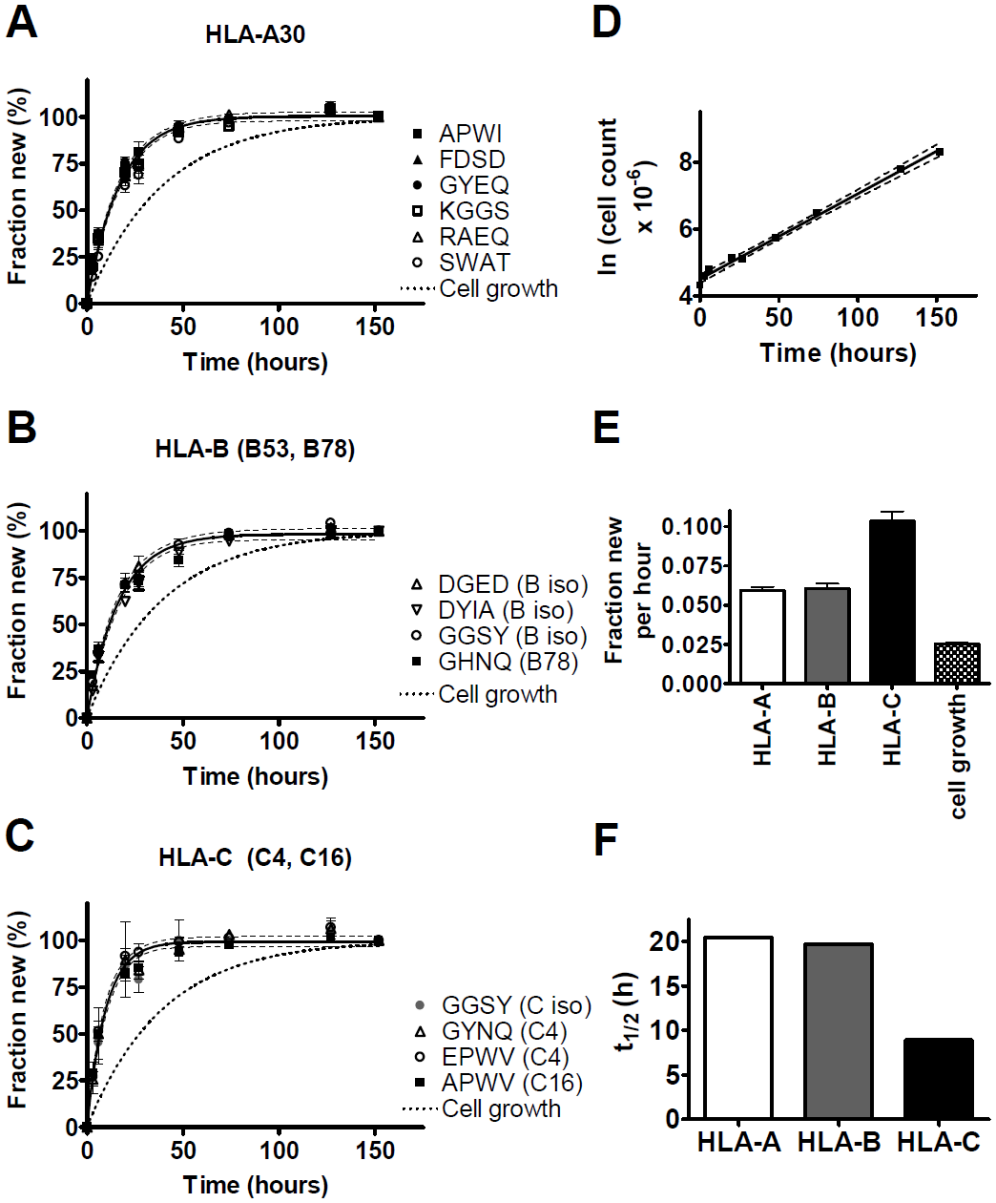
MHCI turnover in KG-1 cells. Proliferating KG-1 cells were labeled with ≈ 5% ^2^H_2_O in media and MHCI molecules immunoprecipitated with W6/32. ^2^H incorporation into selected peptides (S1 Table; identified here by the four N-terminal amino acids and assigned to isotypes and alleles as shown) was quantified by LC-MS. One of two independent experiments is shown here; S1 Table summarizes results for both. (A-C) Fractional synthesis was calculated for different peptides derived from HLA-A (A), HLA-B (B), and HLA-C molecules (C) (mean ± SD of the informative mass isotopomers) and plotted against time. In (B) and (C), allele- and isotype-specific peptides exhibited no significant differences in fractional protein synthesis (p = 0.24 and p = 0.55, respectively, by F test). Single-exponential curve fits (with 95% confidence intervals) are based on a pooled analysis of all peptides from each isotype. (D) Exponential growth of KG-1 cells during ^2^H_2_O labeling. The corresponding time course of the fraction of new cells is shown in panels (A-C) for comparison with protein synthesis. (E) Fractional synthesis rates (per hour, mean ± SEM) of MHCI isotypes (from (A-C)), compared with cell growth (from (D)). The differences between HLA-C and the other isotypes, and those between the MHCI fractional synthesis rates and cell growth, were significant (each p < 0.0001, F test). (F) Turnover half-lives of different MHCI isotypes, calculated from the excess of mean fractional protein synthesis rates over the cell growth rate.

The label incorporation curves for MHCI allele- and isotype-specific peptides in the KG-1 cells are shown in Fig 3(A)–3(C). As expected, no significant differences in fractional protein synthesis were observed between different peptides derived from A30, the only HLA-A allelic variant found in this cell line (Fig 3A). (Note that here and below, the “A30” style is adopted when referring to protein variants, vs. “A*30” for genotypes.) Rate constants for the individual peptides, from two independent experiments, are reported in S1 Table; the best-fit curve in Fig 3A represents a pooled analysis of all informative peptides. No such peptides were found for the B53 allele, but a B78 allele-specific peptide yielded a fractional synthesis rate that was statistically indistinguishable from that for several peptides shared between the two B alleles (Fig 3B). If the two alleles had differed substantially in their fractional synthesis rates, the B78-specific peptide should have diverged from the shared peptides. For the C isotype, peptides specific for C4 and C16, as well as a peptide that was shared between the two alleles, were also indistinguishable (Fig 3C). For all three isotypes, the observed label incorporation curves were modeled well by single-exponential kinetics.

The KG-1 cells maintained steady-state exponential growth throughout this experiment (Fig 3D). The corresponding fraction of new cells is shown in Fig 3(A)–3(C) for comparison with fractional protein synthesis data. The fractional synthesis rates of all three MHCI isotypes substantially exceeded the rate of cell growth (Fig 3E); MHCI levels (by flow cytometry) exhibited only minor fluctuations (not shown), so that the excess of fractional protein synthesis over fractional cell growth represented the replacement of protein lost to turnover. Fractional protein turnover rates calculated on this basis corresponded to half-lives of about 20 hours for HLA-A and -B molecules, and about 9 hours for HLA-C (Fig 3F). There was no evidence of differences between the allelic variants of either HLA-B or -C, and no evidence of kinetic subpopulations within each molecule with different turnover rates.

Growth rates may underestimate the fraction of new cells arising from proliferation, if cell death is also present. In these experiments, however, cell death was negligible, with viability remaining excellent throughout (> 95%). Even if the fraction of new cells had been slightly underestimated, cell death would have removed all MHCI variants at the same rate, and would thus not have confounded comparisons between alleles and isotypes.

Consistent with earlier work [35], the turnover of mature MHCI molecules in these experiments was attributable, at least in part, to lysosomal degradation. Exposure of KG-1 cells to ammonium chloride, a lysosomotropic agent, for several protein half-lives resulted in the gradual accumulation of W6/32-reactive MHCI molecules, mostly in intracellular compartments (S1 Fig).

### Turnover of MHCI molecules in the EBV-B cell line, LCL721

Similarly, MHCI protein turnover was measured by SINEW in LCL721, an EBV-transformed B-lymphoblastoid cell line used in genetic studies of antigen presentation [45, 54–56]. Again, allele- and isotype-specific MHCI HC tryptic fragments were identified by LC-MS/MS and assigned to the MHCI alleles and isotypes present. S2 Table summarizes analytical metrics of all such peptides that proved informative.

**Fig 4:**
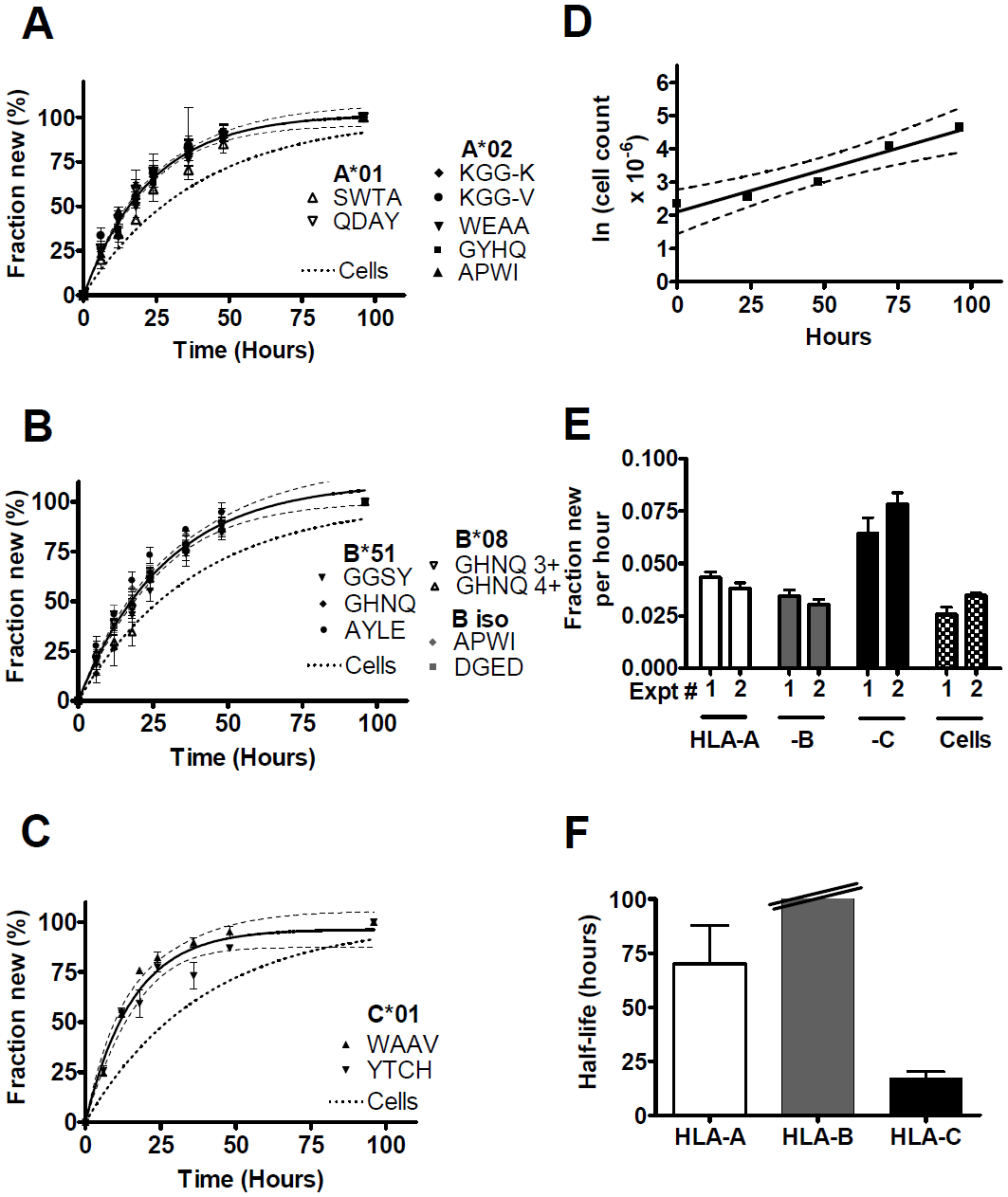
MHCI turnover in LCL721 cells. LCL721 cells were labeled with ≈ 5% ^2^H_2_O in media and folded MHCI molecules immunoprecipitated with W6/32. ^2^H incorporation into tryptic peptides was quantified by LC-MS. (A-C) Fractional synthesis was calculated for different peptides derived from HLA-A (A), HLA-B (B), and HLA-C molecules (C) (mean ± SD of different mass isotopomers) and plotted against labeling time. Full sequences and analytical metrics for all informative peptides (identified by four amino acids in single-letter code or by charge) are in S2 Table. In (A) and (B), allele- and isotype-specific peptides exhibited no significant differences in fractional protein synthesis (p = 0.32 and p = 0.29, respectively, by F test); in (C), only C1-specific peptides were identified. Single-exponential curve fits (with 95% confidence intervals) are based on a pooled analysis of all peptides from each isotype. (D) Exponential growth of LCL721 cells during ^2^H_2_O labeling. The corresponding time course of the fraction of new cells is shown in panels (A-C), for comparison with protein synthesis. Panels (A-D) were from the same experiment. (E) Fractional synthesis rates (per hour, mean ± SEM) of MHCI isotypes (from (A-C)), compared to fractional cell growth rates (from (D)). Two independent experiments are shown. Fractional synthesis rates for individual peptides are in S2 Table. (F) Turnover rates of different MHCI isotypes, calculated from the excess of mean fractional protein synthesis rates over the cell growth rate.

Fractional synthesis curves of HLA-A, -B, and -C molecules in LCL721 are shown in Fig 4(A)–4(C). The fractional synthesis rates of peptides derived from A1 and A2 were statistically indistinguishable from each other (Fig 4A); similarly, the fractional synthesis rates of B8- and B51- specific and B isotype-specific peptides were indistinguishable (Fig 4B). For HLA-C, two peptides, both derived from C1, yielded similar fractional synthesis rates (Fig 4C). MHCI expression levels remained approximately in steady state over time (data not shown). The cell growth rate was determined (Fig 4D) and compared to protein fractional synthesis rates in Fig 4(A)–4(C) and 4(E). The protein synthesis rates were slightly lower than in KG-1 cells, and LCL721 grew faster, so that a smaller proportion of protein synthesis was attributable to protein turnover. For HLA-B, the fractional synthesis rate was very similar to the fractional cell growth rate; for HLA-A, it was slightly larger. The small excess of new HLA-A protein synthesis above this level corresponded to a protein turnover half-life on the order of several days (≈ 65 h), albeit with substantial uncertainty (Fig 4F). For HLA-B, the excess was even smaller, implying an immeasurably slow rate of turnover. For HLA-C1, however, fractional protein synthesis clearly exceeded the amount attributable to cell growth, corresponding to a turnover half-life of ≈ 17 hours (Fig 4C, 4E and 4F).

### Characterization of MoDCs for measurement of MHCI protein turnover

**Fig 5:**
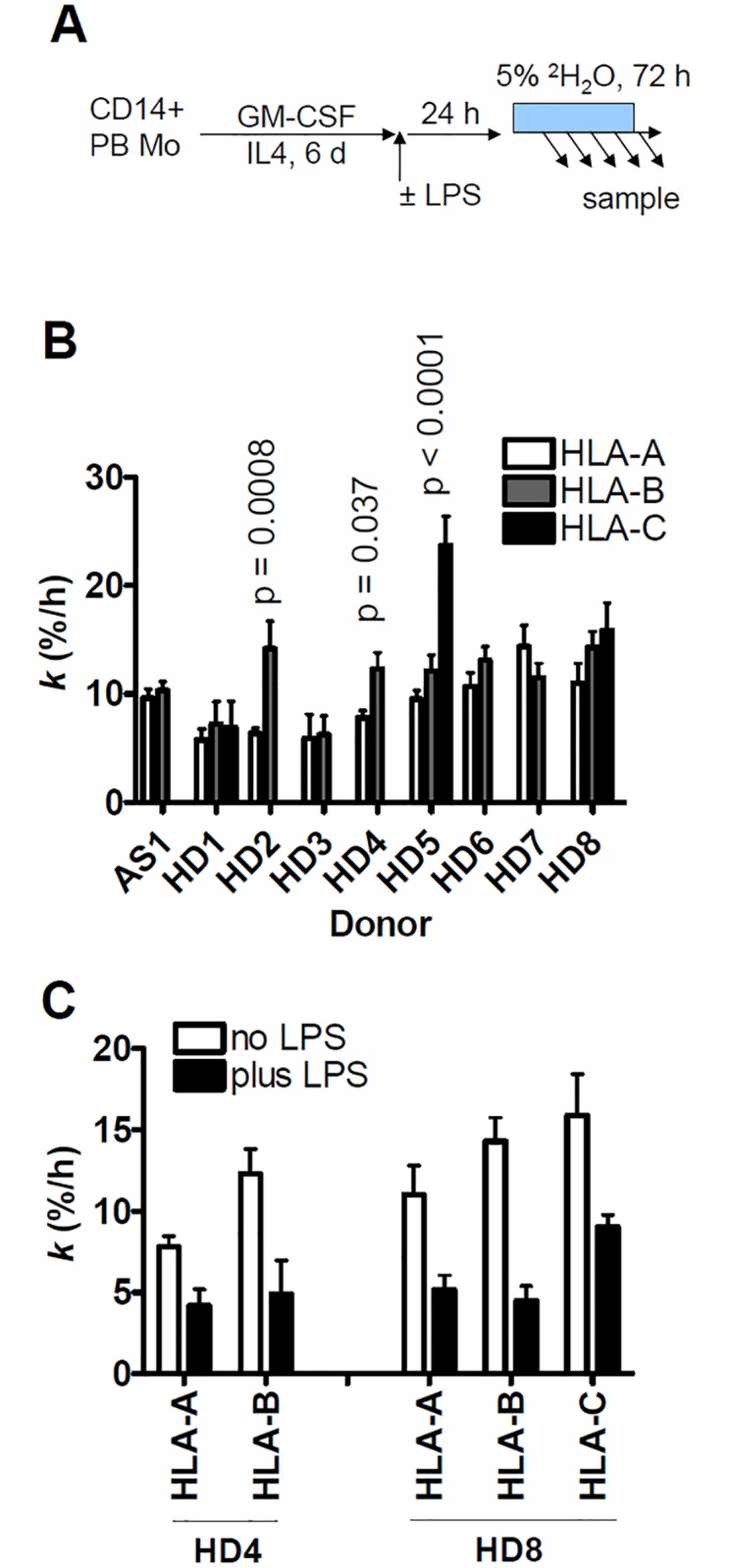
Summary of fractional synthesis rates of MHCI isotypes in MoDCs. (A) Scheme illustrating MoDCs differentiation, followed by mock or LPS (100 ng/ml) stimulation, with subsequent ^2^H_2_O labeling for 72 hours, beginning 24 hours after LPS treatment. (B) Comparison between turnover rates of MHCI isotypes in individual unstimulated MoDC cultures from 8 HDs and one B27-negative AS patient. In this setting, fractional synthesis was taken to equal turnover (see text). (C) Effect of LPS on fractional synthesis rates of MHCI isotypes in two HDs.

In order to examine MHCI protein turnover in non-transformed cells, MoDC cultures were established using standard protocols [46, 47] and used in SINEW experiments (Fig 5A). As expected, after 6 days of culture of CD14-enriched monocytes with IL-4 and GM-CSF, morphology, loss of CD14, and acquisition of CD11c confirmed differentiation to MoDCs. Most of the MoDCs were non-activated at baseline, as indicated by low expression of HLA-DR. Addition of ^2^H_2_O to MoDCs did not alter MHC protein expression. However, the median fluorescence intensities of CD86 (not shown), HLA-DR, and folded MHCI increased severalfold 24 hours after LPS stimulation (representative examples from four HDs shown in S2A and S2B Fig). These levels remained approximately constant during the subsequent 3 days of ^2^H_2_O labelling. Similar numbers of MoDCs were recovered at all time points. In the absence of cell growth or net protein accumulation, protein turnover equals fractional protein synthesis (cf. Fig 1B).

Previous studies had demonstrated that LPS induces a burst of new HLA-DR synthesis, followed by shutdown of DR protein synthesis; existing DR molecules are redistributed to the cell surface, where they persist for extended periods without substantial turnover [57]. ^2^H_2_O labeling of a DRα peptide 24 hours after LPS (or sham) stimulation confirmed the loss of HLA-DR protein turnover. Unstimulated MoDCs showed DR protein synthesis and turnover with half-lives of ≈ 15–25 hours (a representative example is shown in S2C Fig); in contrast, 24 hours after LPS stimulation, virtually no further production of new DR molecules was detectable. Surface DR levels remained high (S2A Fig), so low fractional synthesis implied low turnover (t_1/2_ > 200 hours). We concluded that these MoDC preparations were suitable for measurements of MHC protein turnover, reproducing previously-described differences in MHC protein dynamics between unstimulated and LPS-activated cells [57].

### Allele-independent turnover of MHCI molecules in MoDCs

For measurement of MHCI protein turnover, MoDCs were prepared from eight HDs, including five B*27+ individuals, and one AS patient (who, unusually, was B*27-negative). Folded MHCI molecules were immunoprecipitated with W6/32 and their HCs excised from gels for LC-MS analysis. In all instances, their tryptic fragments were consistent with the donors’ HLA genotypes. The turnover kinetics of the different HLA class I isotypes, and the effect of LPS in two donors, are shown in Fig 5B and 5C, respectively. The sequences, analytical data, and turnover rates of all informative peptides are summarized in S3 Table.

**Fig 6:**
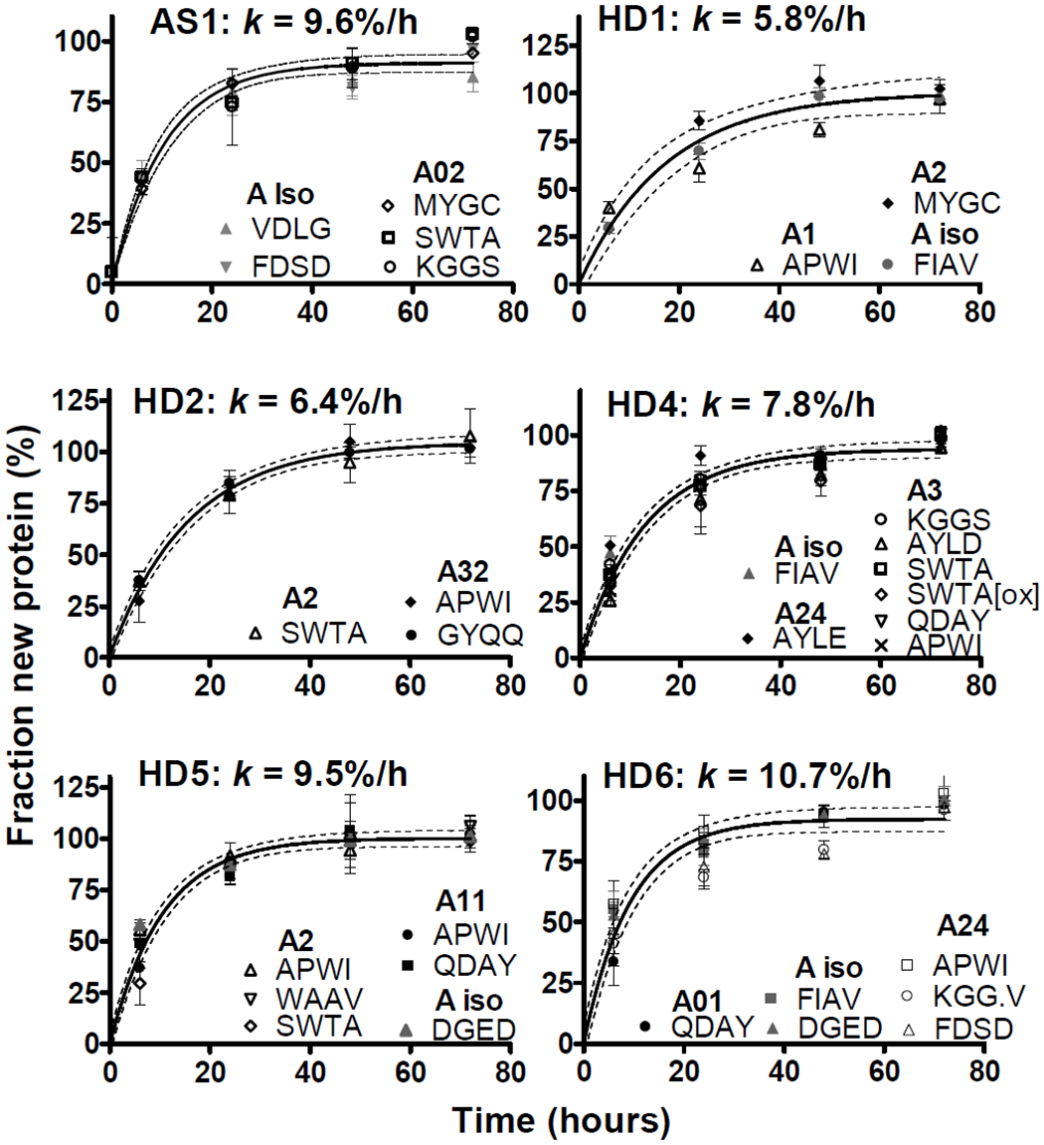
HLA-A fractional synthesis in unstimulated MoDCs. Each panel shows isotype-specific (grey symbols) and allele-specific (black or white symbols) peptides from an individual HD or AS patient. SDs of individual data points are shown, as well best single-exponential curve fits to pooled data, with 95% confidence intervals (dashed lines). Fractional synthesis rate constants (*k*) are shown (means ± SEM).

**Fig 7:**
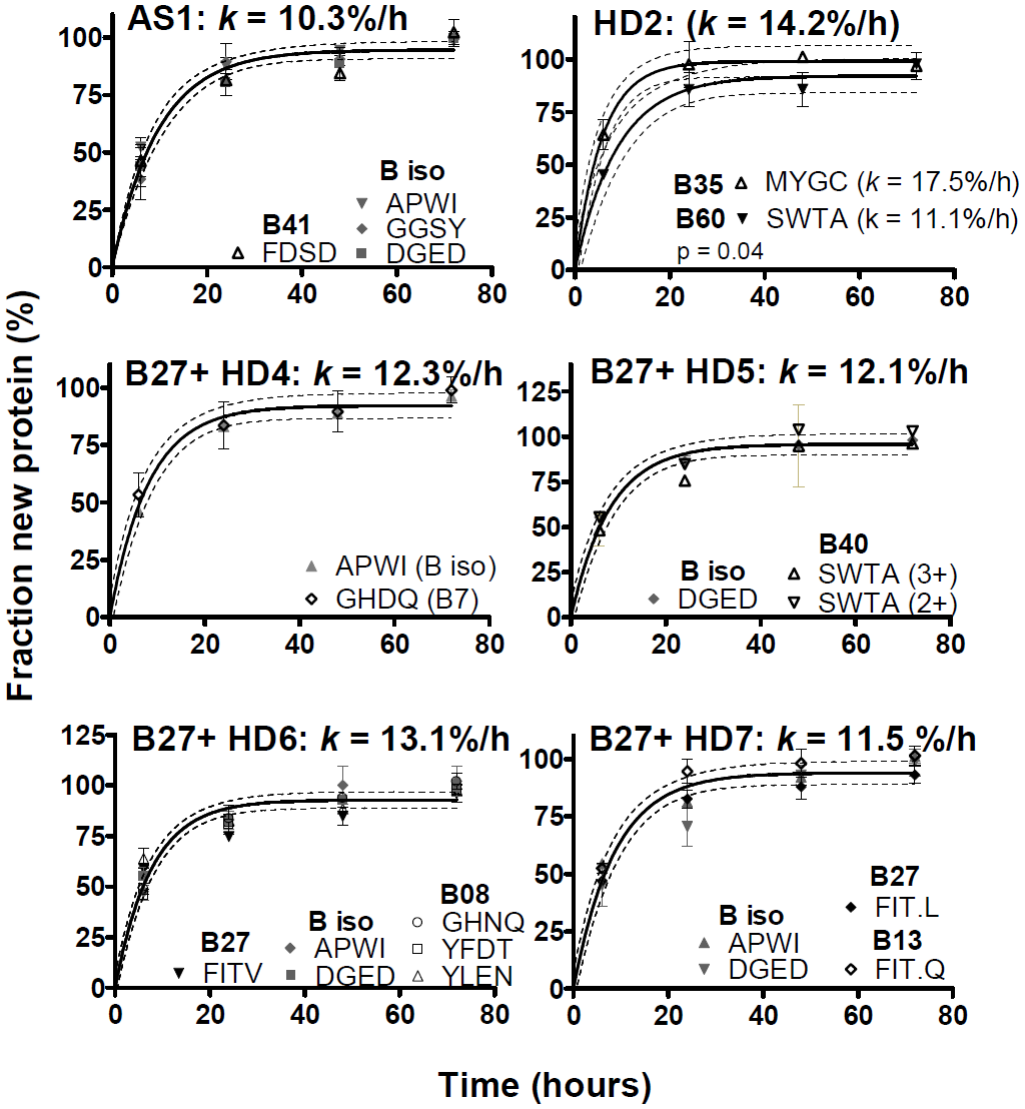
HLA-B fractional synthesis in unstimulated MoDCs. Analysis as in Fig 6, except that separate curve fits are shown for HD2 (p = 0.04, F test). The significance of this result is doubtful, as explained in the text. B*27+ donors are identified; note that B27 allele-specific peptides proved suitable for analysis in HD6 and 7, but not in HD4 and 5.

**Fig 8:**
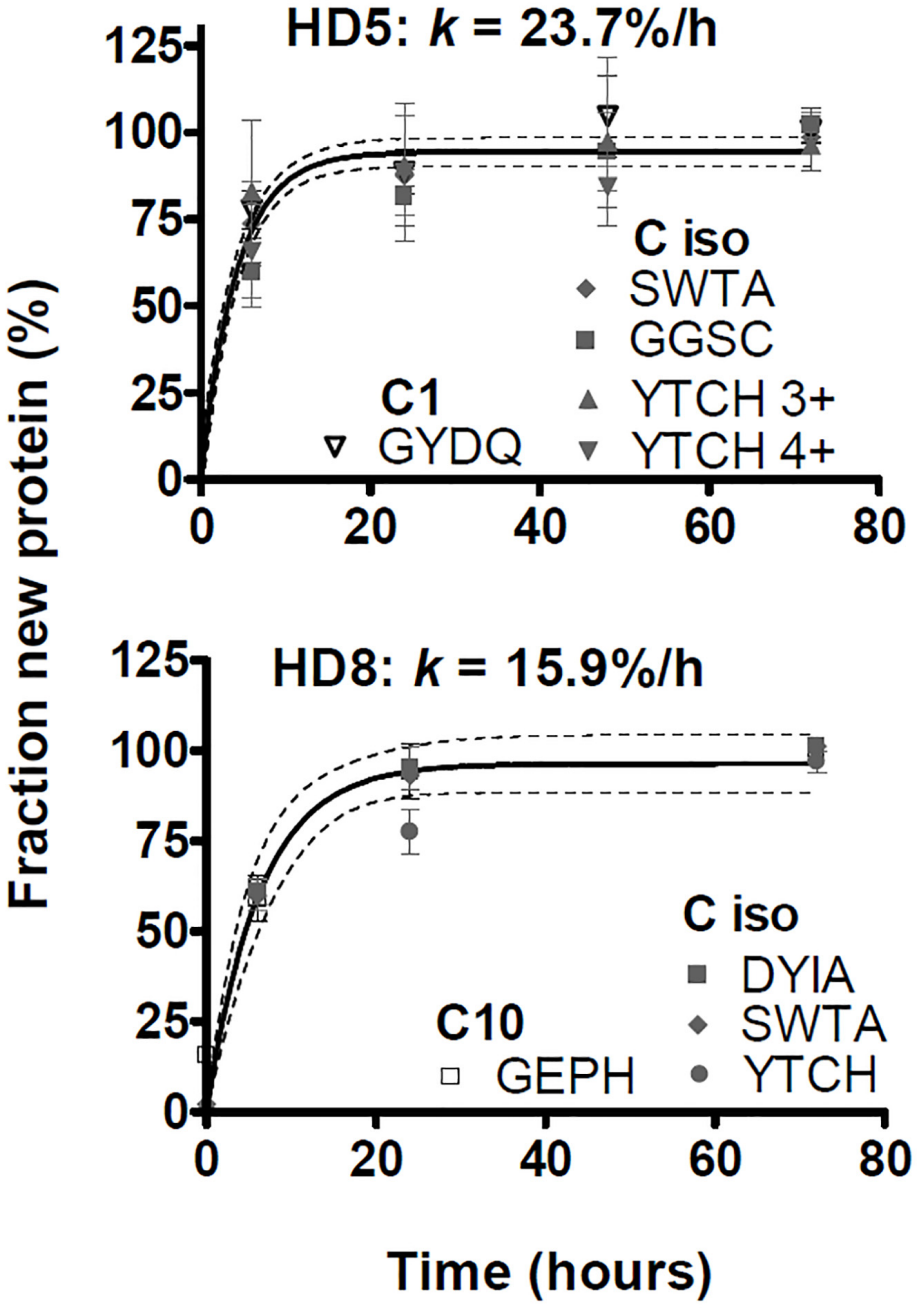
HLA-C fractional synthesis in unstimulated MoDCs. Analysis as in Fig 6.

Fractional synthesis curves comparing allele- with isotype-specific peptides for individual donors are shown in Fig 6 for HLA-A, Fig 7 for HLA-B, and in Fig 8 for HLA-C. Not included in Figs 6–8 were donors in whom the relevant HLA locus was homozygous, or where analysis of allele-specific peptides proved uninformative (the fractional synthesis rates of isotype-specific peptides are included in Fig 5 and S3 Table, however). Peptides specific for the HLA-C isotype only met analytical requirements in three donors, and allele-specific HLA-C peptides only in two donors, likely due to the lower abundance of this isotype [19–21].

The fractional synthesis curves were consistent with a single-exponential rise to plateau, and thus with a uniform turnover rate of folded MHCI molecules (curve fits in Figs 6–8). In all cases except one, the fractional synthesis rates of allele- and isotype-specific peptides from any one donor and locus were not significantly different from each other (p > 0.05, F test; Figs 6–8). Only in HD 2, HLA-B35 and B60 (B*40:01 genotype) turnover rates appeared statistically different from each other (p = 0.04, F test; Fig 7). However, the evidence for this difference was weak: each B allele was represented by just one peptide; the difference was small and within the range of experimental variation observed within other donors; the F test yielded a marginally significant p value, considering that multiple comparisons were made.

The turnover of several HLA-A and -B alleles could be analysed in more than one donor. Five HDs had been recruited for their B*27 genotype, which was therefore over-represented in our sample compared with the general population. B27 allele-specific peptides were tracked directly in two HDs. In three additional donors carrying the B*27 allele, no B27-specific peptides meeting analytical quality criteria were identified, but the other allele exhibited the same labelling kinetics as isotype-specific peptides, consistent with similar turnover rates for B27 and the coexpressed allelic variant. Allele independence was also repeatedly observed for HLA-A2 (in four donors), and for A1 and A24 (in two donors each). To summarize, the results in Figs 6–8 indicate that allelic polymorphism has little or no influence on the turnover rates of folded MHCI molecules in non-activated MoDCs.

Accordingly, the data for allele- and isotype-specific peptides in Figs 6–8 were pooled, improving the estimates of turnover rates of each MHCI isotype shown in Fig 5B. There was tentative evidence that, compared with HLA-B, turnover may have been somewhat slower for HLA-A and faster for HLA-C (where measurable), but this may be donor-dependent: these trends reached the formal threshold for statistical significance (p < 0.05, F test) only in a minority of donors (2/9 for A vs. B, and 1/3 for B vs. C). In most donors, the turnover rates of the three isotypes were similar, averaging on the order of 10%/h (corresponding to t_1/2_ ≈ 7 h). MHCI protein turnover rates in the single, B*27-negative AS patient were typical of the rates measured in the HDs.

Unlike the nearly complete shutdown of HLA-DR turnover by LPS, dendritic cell activation was previously reported to cause a less marked slow-down of MHCI protein turnover [34], or none [57]. To address this, MoDCs from two donors were stimulated with LPS for 24 hours before ^2^H_2_O labeling and analyzed for MHCI protein turnover. An approximately 2-fold slow-down in MHCI turnover was detectable under these conditions (Fig 5B). No allelic differences in turnover rates became apparent following LPS stimulation (S3 Table).

## Discussion

Structural polymorphism, including both allelic and isotypic (locus) variation, influences many aspects of the cellular biochemistry of MHCI molecules, especially in mutant cell lines with defective peptide loading (see Introduction). Thus, we surmised that the turnover rate of folded MHCI protein variants also might be influenced by isotypic and allelic differences in cells with intact peptide loading pathways. However, our recent work in mice had shown that the cellular microenvironment, rather than structural polymorphism, was the main determinant of turnover of MHC class II proteins [49]. Here, we have used a stable isotope tracer technique to compare the turnover rates of classical HLA class Ia alleles, expressed codominantly in host cells with intact peptide loading pathways. Two well-characterised, immortalised model cell lines were used, as well as MoDCs from a small group of volunteers. HLA-A and -B turnover half-lives differed greatly between host cell types, ranging from > 2 days in EBV-transformed B cells to 5 hours in some unstimulated MoDC cultures, and were affected by the activation state of MoDCs (≈ 2-fold slow-down after LPS stimulation). Remarkably, however, the turnover of MHCI molecules was not substantially influenced by allelic polymorphism in any of these settings. In addition, HLA-C exhibited faster turnover than the other two isotypes in the cell lines and in one of the MoDC cultures, but not in the two other donors in whom HLA-C-derived peptides could be analysed. The data have important implications for the mechanisms that determine codominance, the kinetic control of normal antigen presentation and NK cell regulation by MHCI, and the mechanisms linking MHCI polymorphism to autoimmune pathogenesis.

### Kinetic basis of MHCI codominance

Maternal and paternal alleles of the three classical MHCI isotypes, HLA-A, -B, and -C in humans, are codominantly expressed, but our understanding of the determinants of codominance remains incomplete. Several studies have shown isotype differences in post-translational maturation and fate, contributing to the generally lower surface expression of HLA-C, compared to HLA-A and –B, in the steady state [19–21, 30]. The effects of allelic polymorphism are less well known. One early study showed little evidence for allelic disparities in steady-state MHCI protein levels, but acknowledged the limitations of available serological reagents [58]. A few recent studies have shown that non-structural polymorphisms upstream of MHCI genes can control allelic differences in MHCI protein expression [17, 18]. Sometimes a single amino acid change can determine the reliance of MHCI molecules on interactions with the peptide loading complex for maturation, surface expression, and stability, and consequently the fate of MHCI alleles in cells with peptide loading defects [23–27].

Whether MHCI alleles of the same isotype differ in their turnover rates in cells with intact loading pathways, however, had not previously been studied, to our knowledge. Here, we addressed this question by comparing the rates of ^2^H incorporation from ^2^H_2_O between allele- and isotype-specific MHCI-derived tryptic fragments by Orbitrap LC-MS. These experiments indicated that the turnover rates of assembled MHCI molecules in cells with normal peptide loading pathways are largely or entirely allele-independent.

The aggregate evidence for this was extensive. In any one cell line, similar turnover rates were observed by analysis of different allele- and/or isotype-specific peptides, providing internal validation of the data. The lack of allelic differences in turnover was not due to technical limitations, because previously-reported turnover differences were readily resolved by the SINEW method. Specifically, SINEW readily detected a twofold slow-down in MHCI protein turnover following LPS stimulation in MoDC (cf. [34]), and the ≈ twofold faster turnover of HLA-C vs. -A and -B in KG-1 cells (cf. [30]), as well as the almost complete shutdown of HLA-DR turnover following LPS stimulation of MoDCs [57]. Yet, within any one cell culture, allele specific peptides of a given isotype showed similar rates of turnover to one another, or to the appropriate isotype-specific peptides, within narrow limits. Moreover, turnover was allele-independent across a wide range of turnover rates observed in different cell types, which were highest in immature MoDCs and lowest in EBV-transformed B cells. Lastly, our study allowed both a sampling of MHCI polymorphism in the population, as well as repeated analysis of a few allelic variants (A1, A2, A24, and B27) in more than one donor. There was only one example of an allelic difference reaching nominal statistical significance (B alleles in HD2), but this difference was small, based on only one peptide from each allele, and not significant after accounting for multiple comparisons (see Results). In summary, allele-independence was observed for all three MHCI isotypes, across donors, regardless of activation state and host cell lineage, and despite wide variation in the observed rates of MHCI protein turnover.

The allele-independence of MHCI protein turnover in healthy donors suggests that any mismatch between the abundances of codominantly expressed alleles must arise from differences in their relative production rates, e.g. due to disparities in mRNA levels, translation, or post-translational assembly. Published data addressing this point are limited but consistent with this view. For example, HLA-C alleles differ from each other in their relative mRNA abundances, depending on an upstream polymorphism targeted by a micro-RNA; this was shown to correlate with steady-state levels of the C alleles at the cell surface [18]. This supports the idea that any allelic differences in turnover are not large enough to confound difference in production rates.

The fact that, in cells from HDs, folded B27 molecules exhibited similar turnover to those of other B alleles constrains mechanistic models of B27 misfolding, a factor thought to contribute to various mechanisms linking this allotype to genetic risk of spondyloarthritis in transgenic rodent models [59, 60] and in humans [61–63]. Many B*27+ patients with spondyloarthritis also have functionally abnormal ERAP1 variants [64], which are uncommon in healthy donors; whether this affects MHCI protein turnover generally, or B27 turnover specifically, in these patients is an interesting question for future research.

### A role for peptide loading in allele-independent MHCI turnover?

MHCI protein turnover is accelerated in host cells with defective MHCI peptide loading; in the presence of such defects, MHCI turnover rates become allele-dependent, because some alleles are better able than others to acquire an alternative peptide repertoire, remain stable without interaction with the peptide loading complex, escape ERAD, and exit the ER (cf. Introduction). Conversely, one might surmise that acquisition of a stable peptide repertoire, edited by tapasin, is critical for the allele-independent turnover observed here. Stably bound peptides, selected for good fit to allele-specific peptide binding pockets, may diminish the effects of groove polymorphisms on the thermodynamic stability of folded MHCI proteins, and thus equalize their life spans post-Golgi. This would imply that the tapasin-edited peptide repertoire has dissociation half-lives that are at least as long as the turnover rates of folded MHCI molecules in primary cells, or longer. The available data on dissociation half-lives of MHCI-bound peptides *in vitro* are partially, but not uniformly consistent with this [65].

We have not examined whether non-MHC polymorphisms could affect both the rate and allele-dependence of MHCI protein turnover. For example, ERAP2 deficiency, found in about half the human population, is associated with lower steady-state MHCI levels [66]; it is unclear whether this is due to differences in the efficiency of MHCI assembly/maturation, or breakdown, or both. Moreover, structural polymorphisms in ERAP1 may influence peptide trimming, to similar effect, although the great majority of healthy donors have comparable levels of ERAP1 activity [64]. Larger studies of MHCI protein turnover with ERAP-genotyped donors will be required to address this.

### Role of turnover in the low expression of HLA-C

HLA-C has been suggested to be less prominent as a restriction element for CTLs, yet more important in NK cell regulation, than HLA-A or -B [67, 68]. Isotype-dependent differences in turnover rates may, therefore, represent a biochemical adaptation to their different functions. Previous work has shown differences in the internalization rates of HLA-C, vs. HLA-A and –B, in the KG-1 cell line, and mapped these effects to different internalization motifs in the cytoplasmic tails of the three isotypes [30]. This is consistent with the faster turnover rate of HLA-C observed in KG-1 and LCL721, and in one of the MoDC cultures, in the present study. However, data from other MoDC cultures qualify this conclusion. In one culture, HLA-B had atypical turnover, whereas in two other cultures where HLA-C could be analyzed, all three isotypes had approximately equal turnover rates. Individual differences in HLA-C turnover could arise from donor variation in the expression or function of non-MHC genes that determine endosomal trafficking, degradation, or peptide loading. Thus, isotype differences in MHCI turnover rates found in selected model systems may not necessarily be representative of the behaviour of MHCI molecules in the population at large. In conclusion, the low surface expression of HLA-C molecules [19] may be determined by locus differences in gene expression [69] and post-translational maturation [20, 21], with turnover differences making an additional contribution in some, but not all donors.

### Functional significance of MHCI turnover rates

Immune surveillance by effector CTLs in peripheral tissues may be relatively independent of MHCI turnover, because virally infected cells continuously resupply viral peptides for loading of new MHCI molecules. Active turnover of MHCI molecules on bystander cells may, however, help to minimise the risk of killing bystander cells that have transiently acquired exogenous antigenic peptides.

In contrast to CTL effector function, the initiation of CTL responses often relies on the ability of uninfected DCs to cross-present exogenously acquired viral antigens on MHCI molecules. Activated DCs migrate to draining lymph nodes, removing them from the source of antigen; MHCI protein turnover may thus limit the time during which they may prime naïve CD8+ T cells. Based on our data in LPS-activated MoDCs, MHCI protein turnover might become limiting for priming CD8+ T cell responses after a day or so (i.e., after several MHCI protein half-lives). The activation-dependent slow-down in MHCI protein turnover may be helpful in extending the period of antigen encounter, even though, based on studies performed in mice, CD8+ T cells seem to require only brief antigen exposure to maintain subsequent proliferation and effector cell differentiation [70]. This contrasts with the much more sustained antigen presentation enabled by the shutdown of MHC class II protein turnover which, in mice, is required for priming of CD4+ T cells [71].

The limited life span of MHCI molecules in MoDCs may also have implications for the regulation of NK cell effector function. In addition to mechanisms interfering with the fate of existing MHCI molecules, infection with many viruses disrupts new MHCI protein production via shutoff of host gene activity [72, 73]. The rate with which this affects surface levels of MHCI molecules, and thus the speed with which the infected cell becomes sensitive to NK cell lysis, will depend in part on the life span of pre-existing MHCI molecules and the rate with which they are replenished.

In this regard, the very long half-life of MHCI molecules in EBV-transformed B cells is interesting. In these cells, latent EBV infection (type III latency) maintains high levels of MHCI expression [74], perhaps as a means of subverting NK cell responses (in contrast, MHCI expression is lost and NK cell susceptibility increased during lytic infection [73]). The slow MHCI protein turnover in EBV-transformed B cells likely contributes to this phenotype.

### Study limitations

The main limitations of this study relate to its small size, which limited the range of MHCI alleles that we were able to evaluate. Moreover, the study was restricted to MoDCs from HDs and immortalised cell lines under steady-state conditions, precluding evaluation of mechanisms that might operate in other cell types, short-term changes following cellular activation, or the effects of viral infection, autoimmune disease states, or non-MHC genes. Limited analytical sensitivity prevented us from analysing HLA-C protein turnover in more than a few patients. The analysis focussed on folded, W6/32-immunoreactive MHCI proteins, excluding free heavy chains or other conformationally aberrant forms. Lastly, whole-cell extracts were used as a source of MHCI proteins; even though no evidence of kinetic heterogeneity was found, the study left open the possibility that the surface pool of MHCI proteins, which is the critical population of molecules with regard to antigen presentation and NK cell regulation, might have had a somewhat different rate of turnover than the various intracellular pools.

### Concluding remarks

This study has provided a first survey of the turnover rates of folded MHCI alleles in antigen presenting cells (APCs) that have an intact peptide loading pathway and are not genetically manipulated. The turnover rates of folded MHCI molecules were shown to depend markedly on the host cell type, weakly on the activation state of APCs, and in some donors on the MHCI isotype, yet they did not depend significantly on allelic polymorphism. Thus, any quantitative disparities in the codominant expression of MHCI alleles are likely to arise from disparities in MHCI gene transcription, translation, or assembly, rather than turnover. The allelic conservation of MHCI protein turnover may depend on a stably bound peptide repertoire, and may reflect constraints owing to the competing demands of activation of NK cells and CD8+ T cells. Lastly, our data argue against the possibility that pathogenic B27 misfolding arises from an intrinsic instability of folded B27 molecules, but leaves open the possibility of a modifying role for ERAP polymorphisms.

## Supporting Information

S1 Fig Rescue of folded MHCI molecules in KG-1 cells by lysosomotropic agents. Median fluorescence intensities for W6/32 staining are shown for total (A) and cell surface (B) MHCI molecules after up to 3 days of treatment with or without ammonium chloride.
(TIF)

S2 Fig Characterisation of ^2^H_2_O-labeled MoDCs. (A and B) Relative fluorescence intensities (medians) of surface HLA-DR (A) and HLA-A/-B/-C (B) staining in mock-stimulated (open symbols) and LPS-stimulated (closed symbols) MoDCs after varying times of ^2^H_2_O labeling. For each donor, individual data points were expressed relative to the time-averaged MFI value of unstimulated cells (set to 1). LPS stimulation 24 hours before addition of ^2^H_2_O resulted in ≈ 3-fold upregulation of DR and MHCI surface levels, which remained approximately constant throughout the subsequent 72-hour labeling interval. The data shown, from three B27-negative and one B27+ healthy donors, were representative. CD86 upregulation was also observed, as further confirmation of MoDC activation (not shown). (C) LPS-mediated shutdown of HLA-DR protein fractional synthesis (representative example). ^2^H_2_O labeling of DRα was tracked for 72 hours, with or without LPS stimulation 24 hours previously. Fractional synthesis half-lives were ≈ 20 hours without LPS and > 200 h with LPS. The example shown was representative of the MoDC cultures analyzed.
(TIF)

S1 Table Analysis of MHCI-derived tryptic peptides from KG-1 cells. (PDF)

S2 Table Analysis of MHCI-derived tryptic peptides from LCL721 cells. (PDF)

S3 Table Analysis of MHCI-derived tryptic peptides from MoDCs. (PDF)
