# Supplementary figures and images for: Allele-Independent Turnover of Human Leukocyte Antigen (HLA) Class Ia Molecules
Source: PLoS One. 2016 Aug 16;11(8):e0161011. doi: 10.1371/journal.pone.0161011 (PMC4987023; doi:10.1371/journal.pone.0161011)

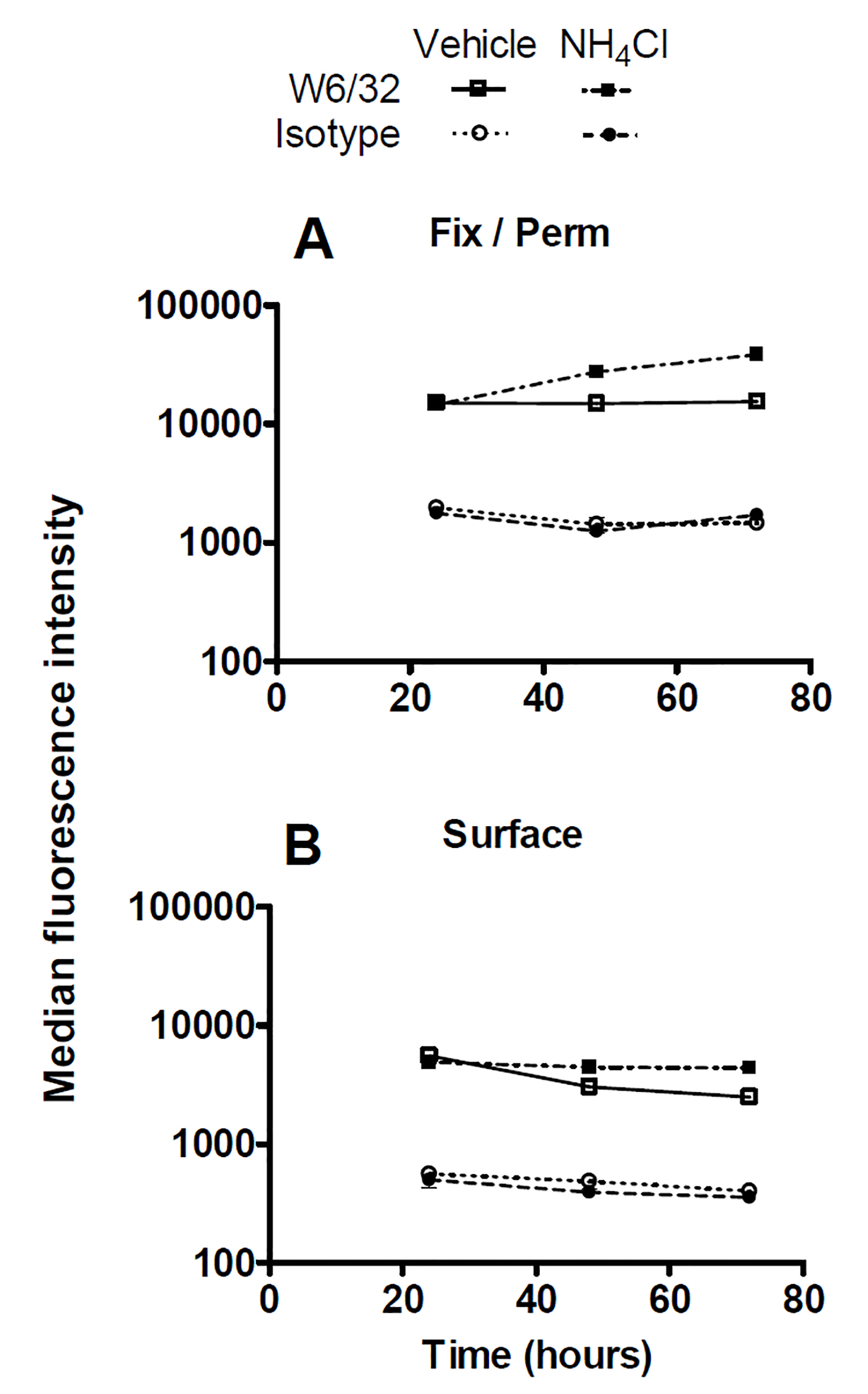

Supplement: S1 Fig — Median fluorescence intensities for W6/32 staining are shown for total (A) and cell surface (B) MHCI molecules after up to 3 days of treatment with or without ammonium chloride. (TIF) [file pone.0161011.s001.tif]

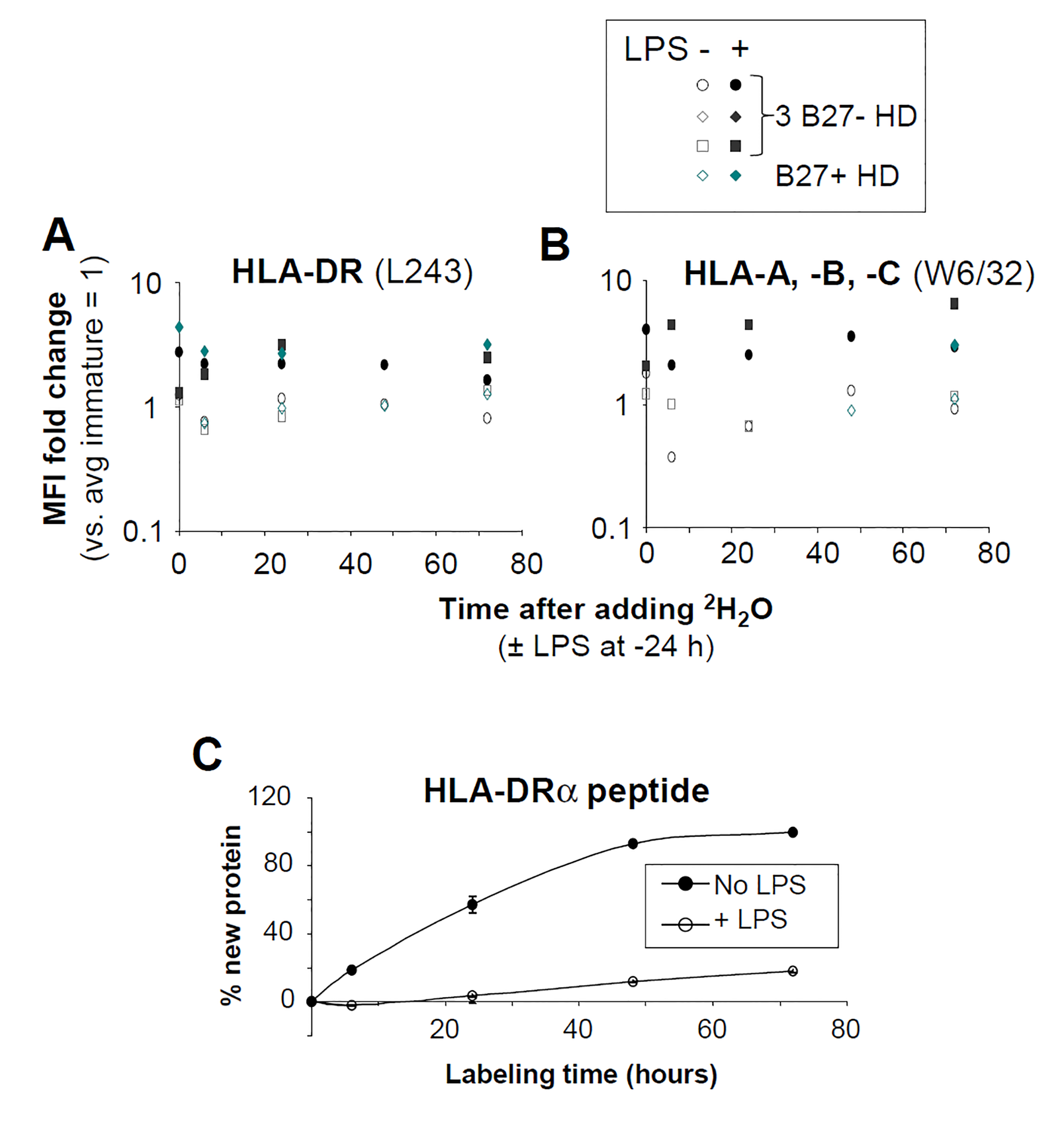

Supplement: S2 Fig — (A and B) Relative fluorescence intensities (medians) of surface HLA-DR (A) and HLA-A/-B/-C (B) staining in mock-stimulated (open symbols) and LPS-stimulated (closed symbols) MoDCs after varying times of 2H2O labeling. For each donor, individual data points were expressed relative to the time-averaged MFI value of unstimulated cells (set to 1). LPS stimulation 24 hours before addition of 2H2O resulted in ≈ 3-fold upregulation of DR and MHCI surface levels, which remained approximately constant throughout the subsequent 72-hour labeling interval. The data shown, from three B27-negative and one B27+ healthy donors, were representative. CD86 upregulation was also observed, as further confirmation of MoDC activation (not shown). (C) LPS-mediated shutdown of HLA-DR protein fractional synthesis (representative example). 2H2O labeling of DRα was tracked for 72 hours, with or without LPS stimulation 24 hours previously. Fractional synthesis half-lives were ≈ 20 hours without LPS and > 200 h with LPS. The example shown was representative of the MoDC cultures analyzed. (TIF) [file pone.0161011.s002.tif]
