# Supplementary material for: Allele-Independent Turnover of Human Leukocyte Antigen (HLA) Class Ia Molecules
Source: PLoS One. 2016 Aug 16;11(8):e0161011. doi: 10.1371/journal.pone.0161011 (PMC4987023; doi:10.1371/journal.pone.0161011)
Supplement: S1 Table — (PDF) [file pone.0161011.s003.pdf]

S1 Table. Analysis of MHCI-derived tryptic peptides from KG-1 cells.

						**Experiment 1** (p = 4.2%) [a]						**Experiment 2** (p = 4.9%) [a, d]					
						**Unlabeled**		**Fully labeled**			**FSR (%/h, mean ± SEM)** [f]	**Unlabeled**		**Labeled**			**FSR (%/h, mean ± SEM)** [f]
**Molecule**	**Sequence** [b]	**Residues** [c]	**Calculated *m/z***	**Observed *m/z***	***z***	**Abundance**	**RMSD**	**Abundance**	**RMSD**	**n** [e]		**Abundance**	**RMSD**	**Abundance**	**RMSD**	**n** [e]	
HLA-A30	FDSDAASQR	36-44	498.72	498.72	2	2.79E+07	0.18%	5.30E+07	0.07%	13	5.59 ± 0.56	1.39E+08	0.25%	7.87E+07	0.35%	13	6.22 ± 0.41
	GYEQHAYDGK	112-121	584.26	584.26	2	7.11E+06	1.80%	5.95E+06	0.47%	12	6.21 ± 0.64	8.92E+06	0.47%	8.00E+06	0.52%	11	7.08 ± 0.57
	RAEQLR	152-157	386.73	386.73	2	2.93E+07	0.92%	6.88E+07	0.57%	7	5.29 ± 0.45	1.41E+08	0.46%	7.09E+07	0.42%	7	5.67 ± 0.46
	SWATAADM[ox]AAQITQR	132-145	783.38	783.38	2	2.23E+07	0.92%	3.98E+07	0.16%	16	5.84 ± 0.74	2.25E+07	0.14%	5.23E+07	0.31%	18	4.50 ± 0.22
	APWIEQERPEYWDQETR	49-65	745.02	745.02	2	5.71E+07	0.52%	1.30E+08	0.37%	25	7.71 ± 0.58	2.06E+08	2.19%	1.71E+08	0.27%	30	6.69 ± 0.32
	KGGSYTQAASSDSAQGSDVSLTACK	316-340	826.05	826.05	3							1.62E+07	0.53%	3.52E+07	0.24%	26	6.01 ± 0.49
HLA-B iso	DGEDQTQDTELVETRPAGDR	220-239	744.68	744.68	2	3.37E+08	0.36%	3.70E+08	0.23%	32	8.19 ± 0.33	2.06E+08	0.34%	1.68E+08	0.22%	32	6.68 ± 0.32
	DYIALNEDLSSWTAADTAAQITQR	122-145	885.10	885.10	3	1.96E+07	0.30%	7.67E+07	0.20%	31	6.43 ± 0.37	3.95E+06	0.20%	2.64E+07	0.25%	33	5.21 ± 0.32
	GGSYSQAASSDSAQGSDVSLTA	317-338	1023.45	1023.45	2	3.30E+07	0.15%	5.75E+07	0.35%	26	6.58 ± 0.66	1.60E+07	0.47%	1.02E+07	0.40%	27	5.96 ± 0.44
HLA-B78	GHNQYAYDGK	112-121	576.76	576.76	2	1.94E+07	0.18%	1.85E+07	0.62%	8	5.89 ± 0.57	1.74E+07	0.31%	1.40E+07	0.39%	10	6.28 ± 0.77
HLA-B53	(none found)																
HLA-C iso	GGSCSQAASSNSAQGSDESLIACK	318-341	1186.52	1186.52	2	1.88E+06	0.95%	4.18E+06	0.25%	29	7.05 ± 1.12	7.88E+06	0.22%	8.56E+06	0.14%	31	9.09 ± 1.09
HLA-C4	EPWVEQEGPEYWDR	49-62	910.40	910.40	2							5.20E+06	1.21%	2.39E+07	0.87%	21	11.1 ± 0.67
	GYNQFAYDGK	112-121	581.76	581.76	2							3.50E+06	0.67%	7.57E+06	0.03%	9	10.3 ± 1.20
HLA-C16	GYDQSAYDGK	112-121	552.24	552.24	2	2.83E+06	0.49%	7.90E+06	0.62%	12	10.2 ± 1.28						
	APWVEQEGPEYWDR	49-62	881.40	881.40	2							1.61E+07	0.34%	7.15E+07	0.38%	23	10.8 ± 1.00
	Cell growth rate										1.90 ± 0.01						2.55 ± 0.07

Notes:

[a] ^2H_2O enrichment in media (average of samples), used as precursor pool enrichment (p) in MIDA models.

[b] Peptides were selected for allele- or isotype specificity, as described in text, and filtered to meet analytical criteria as described in Methods. Single-letter code ("ox" is M oxidation). The four N-terminal amino acids were used to identify the peptides in Fig. 3.
Note that different peptides met analytical requirements in the two experiments (blanks = not detected or not suitable for analysis), yet fractional synthesis rates remained similar.

[c] Numbering from 1 = N terminus of mature polypeptide as specified in the IMGT/HLA database.

[d] Results from Experiment 2 are shown in Fig. 3.

[e] Number of labeling sites used in MIDA models. RMSD values are for deviations between modeled and observed mass isotopomer distributions.

[f] Fractional synthesis rate (per cent new protein per hour) calculated from curve fits for individual peptides. Peptides from the same allele or isotype yielded statistically indistinguishable values from one another (p > 0.05, F test), so pooled data were used in Fig. 3.
