# Supplementary material for: Allele-Independent Turnover of Human Leukocyte Antigen (HLA) Class Ia Molecules
Source: PLoS One. 2016 Aug 16;11(8):e0161011. doi: 10.1371/journal.pone.0161011 (PMC4987023; doi:10.1371/journal.pone.0161011)
Supplement: S2 Table — (PDF) [file pone.0161011.s004.pdf]

S2 Table. Analysis of MHCI-derived tryptic peptides from LCL721 cells.

							Experiment 1 (p = 5.11%) [a]						Experiment 2 (p = 4.50%) [a]					
							Unlabeled		Labeled				Unlabeled		Labeled			
Molecule	Peptide Sequence [b]	Residues [c]	Notation [d]	Calculated *m/z*	Observed *m/z*	*z*	Abundance	RMSD	Abundance	RMSD	n [e]	FSR (%/h) [f]	Abundance	RMSD	Abundance	RMSD	n [e]	FSR (%/h) [f]
HLA-A1	SWTAADM*AAQITK *[ox] in Expt 2	132-144	SWTA	697.34 / 705.34*	697.33 / 705.22*	2	1.11E+08	0.63%	1.14E+08	0.51%	11	3.24 ± 0.20	1.59E+07	1.48%	4.81E+07	0.39%	15	2.49 ± 0.30
	FDSDAASQK	36-44		484.72	484.66	2							2.28E+07	0.82%	1.04E+08	0.38%	13	3.94 ± 0.24
	QDAYDGKDYIALNEDLR	115-131	QDAY	666.99	666.97	3	4.65E+08	0.38%	5.08E+08	0.17%	20	4.83 ± 0.14						
	APWIEQEGPEYWDQETR	49-65		1067.480	1,067.40	2							1.17E+08	1.50%	2.17E+08	0.40%	23	5.34 ± 0.22
HLA-A2	KGGSYSQAASSDSAQGSDVSLTACK	316-340	KGG-K	821.38	821.37	3	8.26E+07	0.76%	1.10E+08	0.84%	17	4.09 ± 0.34						
	KGGSYSQAASSDSAQGSDVSLTACKV	316-341	KGG-V	854.40	854.40	3	2.50E+07	0.87%	7.41E+07	0.49%	19	4.94 ± 0.41						
	GYHQYAYDGKDYIALK	112-127	GYHQ	476.99	476.99	4	1.58E+08	0.43%	2.32E+08	0.49%	13	4.07 ± 0.23						
	WEAAHVAEQLR	147-157	WEAA	437.23	437.23	3	1.13E+08	2.22%	1.04E+08	0.31%	11	4.28 ± 0.29						
	APWIEQEGPEYWDGETR	49-65	APWI	688.31	688.30	3	2.59E+08	0.50%	2.49E+08	0.40%	20	5.16 ± 0.20						
	FDSDAASQR	49-65		498.72	498.62	2							3.13E+07	0.60%	1.11E+08	0.53%	12	3.88 ± 0.10
HLA-B8	GHNQYAYDGKDYIALNEDLR [g]	112-131	GHNQ 4+	589.53	589.53	4	4.92E+08	0.92%	2.98E+08	0.21%	19	3.10 ± 0.51						
	GHNQYAYDGKDYIALNEDLR [g]	112-131	GHNQ 3+	785.71	785.70	3	1.33E+08	1.02%	1.09E+08	0.59%	19	3.65 ± 0.19						
HLA-B51	AYLEGLCVEWLR	158-169	AYLE	754.88	754.87	2	6.77E+08	1.42%	4.61E+08	0.44%	10	5.03 ± 0.19	1.56E+08	0.49%	4.10E+08	0.31%	12	3.76 ± 0.39
	GHNQYAYDGKDYIALNEDLSSWTAADTAAQITQR	112-145	GHNQ	947.45	947.44	4	2.15E+08	0.37%	1.94E+08	0.70%	24	3.36 ± 0.52						
	GGSYSQAASSDSAQGSDVSLTA	317-338	GGSY	1,023.45	1,023.44	2	7.85E+07	0.30%	7.41E+07	0.85%	15	2.94 ± 0.22	6.49E+07	0.58%	6.98E+07	0.59%	24	2.25 ± 0.15
	DYIALNEDLSSWTAADTAAQITQR	122-145		885.10	885.259	3							7.70E+07	0.97%	3.59E+08	0.60%	29	2.32 ± 0.23
HLA-B iso	DGEDQTQDTELVETRPAGDR	220-239	DGED	1,116.50	1,116.49	2	2.52E+08	0.48%	1.89E+08	0.18%	24	3.85 ± 0.16						
	APWIEQEGPEYWDR	49-62	APWI	888.41	888.39	2	1.82E+09	1.91%	1.48E+09	0.28%	17	4.55 ± 0.22	5.23E+08	0.14%	1.08E+09	0.36%	21	3.79 ± 0.22
HLA-C1	WAAVM[ox]VPSGEEQR	244-256	WAAV	738.34	738.34	2	9.85E+07	0.21%	7.00E+07	0.82%	14	6.56 ± 0.64						
	YTCHVQHEGLPEPLTLR	257-273	YTCH	684.01	684.01	3	6.03E+07	1.12%	2.69E+07	0.85%	13	5.88 ± 0.87						
HLA-C iso	APWVEQEGPEYWDR	49-62		881.40	881.320	2							5.58E+07	0.99%	1.12E+08	0.33%	19	7.82 ± 0.55

Notes:

[a] ^2H_2O enrichment in media (average of samples), used as precursor pool enrichment (p) in MIDA models.

[b] Peptides were selected for allele- or isotype specificity, as described in text, and filtered to meet analytical criteria as described in Methods. Single-letter code ("ox" refers to M oxidation)
Note that different peptides met analytical requirements in the two experiments (blanks = not detected or not suitable for analysis), yet fractional synthesis rates remained similar.

[c] Numbering from 1 = N terminus of mature polypeptide as specified in the IMGT/HLA database.

[d] Used in Fig 4 legend, where the results from Experiment 1 are shown in (A)-(C).

[e] Number of labeling sites used in MIDA models. RMSD values are for deviations between modeled and observed mass isotopomer distributions.

[f] Fractional synthesis rate (per cent new protein per hour). Peptides from the same allele or isotype yielded statistically indistinguishable values from one another ($p > 0.05$, F test). The pooled analysis is shown in Fig. 4.
