# Supplementary material for: Allele-Independent Turnover of Human Leukocyte Antigen (HLA) Class Ia Molecules
Source: PLoS One. 2016 Aug 16;11(8):e0161011. doi: 10.1371/journal.pone.0161011 (PMC4987023; doi:10.1371/journal.pone.0161011)
Supplement: S3 Table — (PDF) [file pone.0161011.s005.pdf]

Table S3. Analysis of MHCI-derived peptides from MoDCs.

**AS1** [A2, 3; B8, 41; C7, 17] [a]												
						**Unlabeled**		**72 h labeled** (p = 4.75%) [e]				
**Molecule**	**Residues**	**Sequence** [b]	***m/z* (pred)** [c]	***m/z* (obs)**	***z***	**Abundance**	**RMSD (%)** [d]	**Abundance**	**RMSD (%)**	**n**	***k* (%/h)** [f]	k_{iso} **(%/h)**
A2	98-108	MYGCDVGSDWR	673.27	673.27	2	2.00E+07	0.41%	1.42E+07	0.40%	7	9.09 ± 1.15	
A2	132-144	SWTAADMAAQTTK	691.32	691.33	2	3.99E+07	0.68%	2.20E+07	0.35%	9	7.89 ± 2.07	
A2	316-341	KGGSYSQAASSDSAQGSDVSLTACKV	854.40	854.40	3	6.94E+06	0.29%	1.16E+07	0.17%	14	7.88 ± 2.08	9.62 ± 0.83
A iso	76-82	VDLGTLR	387.23	387.23	2	1.57E+08	0.61%	1.13E+08	1.55%	5	12.1 ± 0.85	
A iso	36-44	FDSDAASQR	498.72	498.72	2	4.81E+06	0.43%	1.33E+06	0.48%	9	10.9 ± 2.41	
B41	36-44	FDSDATSPR	498.23	498.23	2	2.09E+07	4.84%	6.05E+05	0.48%	10	10.6 ± 3.24	
B iso	49-62	APWIEQEGPEYWDR	888.41	888.40	2	6.19E+07	0.17%	4.88E+07	0.38%	15	12.8 ± 1.15	10.3 ± 0.86
B iso	220-239	DGEDQTQDTELVETRPAGDR	1116.50	1116.50	2	3.28E+06	0.37%	2.77E+06	0.76%	25	9.49 ± 1.61	
B iso	317-338	GGSYSQAACSDSAQGSDVSLTA	1059.95	1059.95	2	1.09E+07	0.46%	7.42E+06	0.27%	13	9.01 ± 1.24	

**HD1** [A1, 2; B37, 49; Cw6, w7] [a]												
						**Unlabeled**		**72 h labeled** (p = 4.93%) [e]				
**Molecule**	**Residues**	**Sequence** [b]	***m/z* (pred)** [c]	***m/z* (obs)**	***z***	**Abundance** [g]	**RMSD (%)** [d]	**Abundance**	**RMSD (%)**	**n**	***k* (%/h)** [f]	k_{iso} **(%/h)**
A1	49-65	APWIEQEGPEYWDQETR	1067.48	1067.48	2	*1.17E+07*		2.25E+07	1.28%	20	5.78 ± 2.62	
A2	98-108	MYGCDVGSDWR	673.27	673.27	2	*5.95E+06*		1.82E+07	0.97%	5	6.17 ± 1.04	5.75 ± 1.00
A iso	22-35	FIAVGYVDDTQFVR	815.42	815.41	2	*1.01E+08*		1.42E+08	0.14%	7	5.23 ± 0.81	
B iso	49-62	APWIEQEGPEYWDR	888.41	888.40	2	*9.29E+06*		3.27E+07	0.94%	13	7.23 ± 2.06	7.23 ± 2.06
C iso	132-145	SWTAADTAAQITQR	760.38	760.38	2	*2.25E+06*		5.44E+06	1.10%	9	6.90 ± 2.43	6.90 ± 2.43

**HD2** [A2, 32; B35, 60 *(*40:01)* ; Cw4, w10 *(*03:04)*] [a]												
						**Unlabeled**		**72 h labeled** (p = 4.98%) [e]				
**Molecule**	**Residues**	**Sequence** [b]	***m/z* (pred)** [c]	***m/z* (obs)**	***z***	**Abundance** [g]	**RMSD (%)** [d]	**Abundance**	**RMSD (%)**	**n**	***k* (%/h)** [f]	k_{iso} **(%/h)**
A2	132-144	SWTAADM[ox]AAQTTK	699.32	699.33	2	*2.49E+06*		2.07E+06	0.92%	8	6.35 ± 1.11	
A32	49-65	APWIEQEGPEYWDQETR	1067.48	1067.48	2	*4.68E+06*		4.36E+06	0.58%	17	5.50 ± 0.84	6.37 ± 0.46
A32	112-131	GYQQDAYDGKDYIALNEDLR	783.03	783.04	3	*2.16E+07*		1.11E+07	0.45%	21	7.51 ± 0.11	
B35	98-108	MYGCDLGPDGR	620.76	620.76	2	*9.54E+06*		1.00E+07	0.41%	7	17.5 ± 1.12	(14.2 ± 2.52)
B60	132-145	SWTAADTAAQISQR	753.37	753.37	2	*1.89E+07*		5.10E+06	0.37%	11	11.1 ± 2.13	(p = 0.039)

**HD3** [A2, 24; B27, 51; C14, 15] [a]												
						**Unlabeled**		**72 h labeled** (p = 4.16%) [e]				
**Molecule**	**Residues**	**Sequence** [b]	***m/z* (pred)** [c]	***m/z* (obs)**	***z***	**Abundance** [g]	**RMSD (%)** [d]	**Abundance**	**RMSD (%)**	**n**	***k* (%/h)** [f]	k_{iso} **(%/h)**
A iso	158-169	AYLEGTCVDGLR	677.33	677.33	2	*7.75E+06*		5.60E+06	0.69%	8	5.89 ± 2.21	5.89 ± 2.21
B iso	49-62	APWIEQEGPEYWDR	888.41	888.40	2	*8.21E+06*		1.62E+07	0.28%	15	6.24 ± 1.69	6.24 ± 1.69

**HD4** [A3, 24; B7, 27; Cw2, w7] [a]												
						**Unlabeled**		**72 h labeled** (p = 5.08%) [e]				
**Molecule**	**Residues**	**Sequence** [b]	***m/z* (pred)** [c]	***m/z* (obs)**	***z***	**Abundance** [g]	**RMSD (%)** [d]	**Abundance**	**RMSD (%)**	**n**	***k* (%/h)** [f]	k_{iso} **(%/h)**
A3	316-341	KGGSYTQAASSDSAQGSDVSLTACKV	859.07	859.08	3	*1.10E+07*		2.53E+06	0.21%	10	8.47 ± 1.61	
A3	158-169	AYLDGTCVEWLR	741.86	741.86	2	*4.11E+07*		6.52E+06	0.48%	8	5.61 ± 0.86	
A3	132-144	SWTAADMAAQITK	697.34	697.34	2	*2.96E+07*		4.96E+06	0.32%	7	7.43 ± 1.49	
A3	132-144	SWTAADM[ox]AAQITK	705.34	705.34	2	*2.36E+07*		3.62E+06	0.74%	8	6.01 ± 1.85	
A3	115-131	QDAYDGKDYIALNEDLR	666.99	666.99	3	*6.15E+07*		1.02E+07	0.30%	17	7.19 ± 1.00	7.80 ± 0.65
A3	49-65	APWIEQEGPEYWDQETR	1067.48	1067.48	2	*1.30E+07*		3.45E+06	0.28%	17	6.35 ± 1.18	
A24	158-169	AYLEGTCVDGLR	677.33	677.33	2	*2.91E+07*		6.56E+06	0.19%	7	12.3 ± 2.27	
A iso	22-35	FIAVGYVDDTQFVR	815.42	815.42	2	*8.99E+07*		3.76E+07	0.60%	7	11.1 ± 2.30	
B7	112-131	GHDQYAYDGKDYIALNEDLR	786.03	786.03	3	3.42E+07		3.49E+06	0.49%	16	13.6 ± 2.82	12.3 ± 1.53

Molecule	Residues	Sequence [b]	*m/z* (pred) [c]	*m/z* (obs)	*z*	Unlabeled Abundance [g]	Unlabeled RMSD (%) [d]	72 h labeled Abundance	72 h labeled RMSD (%)	n	*k* (%/h) [f]	k_{iso} (%/h)
B iso	49-62	APWIEQEGPEYWDR	888.41	888.40	2	*1.16E+08*		4.09E+07	0.40%	13	11.2 ± 1.53	[illegible]

**HD4** [A3, 24; B7, 27; Cw2, w7] [a] **(LPS-stimulated)**												
						**Unlabeled**		**72 h labeled** (p = 5.02%) [e]				
**Molecule**	**Residues**	**Sequence** [b]	***m/z* (pred)** [c]	***m/z* (obs)**	***z***	**Abundance** [g]	**RMSD (%)** [d]	**Abundance**	**RMSD (%)**	**n**	***k* (%/h)** [f]	**k_{iso} (%/h)**
A3	316-341	KGGSYTQAASSDSAQGSDVSLTACKV	859.07	859.08	3	*1.08E+07*		6.07E+07	0.53%	10	4.41 ± 3.22	4.20 ± 0.99
A3	158-169	AYLDGTCVEWLR	741.86	741.86	2	*4.11E+07*		1.73E+08	0.58%	6	2.95 ± 3.53	
A3	132-144	SWTAADM[ox]AAQITK	705.34	705.34	2	*2.34E+07*		6.59E+07	0.74%	7	2.94 ± 4.67	
A3	115-131	QDAYDGKDYIALNEDLR	666.99	666.99	3	*5.75E+07*		1.89E+08	0.50%	15	4.55 ± 1.43	
A3	49-65	APWIEQEGPEYWDQETR	1067.48	1067.48	2	*1.79E+07*		1.31E+08	0.53%	16	3.91 ± 2.18	
A24	158-169	AYLEGTCVDGLR	677.33	677.33	2	*2.90E+07*		1.55E+08	0.30%	7	5.75 ± 7.69	
A24	316-341	KGGSYSQAASSDSAQGSDVSLTACKV	854.40	854.40	3	*4.25E+06*		4.93E+07	0.45%	11	6.02 ± 3.36	
A iso	22-35	FIAVGYVDDTQFVR	815.42	815.42	2	*8.90E+07*		6.20E+08	0.26%	6	4.45 ± 2.34	
B27	22-35	FITVGYVDDTLFVR	822.94	822.93	2	*7.40E+07*		6.21E+08	0.08%	5	4.95 ± 4.83	4.91 ± 2.06
B iso	49-62	APWIEQEGPEYWDR	888.41	888.40	2	*1.16E+08*		1.04E+09	0.13%	12	4.86 ± 1.58	

**HD5** [A2, 11; B27, 60 *(*40:01)* ; Cw1, w10 *(*03:02)*] [a]												
						**Unlabeled**		**72 h labeled** (p = 4.78%) [e]				
**Molecule**	**Residues**	**Sequence** [b]	***m/z* (pred)** [c]	***m/z* (obs)**	***z***	**Abundance** [g]	**RMSD (%)** [d]	**Abundance**	**RMSD (%)**	**n**	***k* (%/h)** [f]	**k_{iso} (%/h)**
A2	49-65	APWIEQEGPEYWDGETR	688.31	688.31	3	*8.29E+06*		5.61E+06	0.43%	17	13.9 ± 1.68	9.51 ± 0.78
A2	244-256	WAAVVVPSGQEQR	713.88	713.88	2	*4.63E+07*		9.79E+06	0.53%	6	9.39 ± 1.85	
A2	132-144	SWTAADMAAQTTK	699.32	699.32	2	*4.61E+06*		2.45E+06	0.40%	7	7.01 ± 1.30	
A11	49-65	APWIEQEGPEYWDQETR	1067.48	1067.48	2	*1.12E+07*		2.88E+06	0.53%	16	6.92 ± 0.61	
A11	115-131	QDAYDGKDYIALNEDLR	666.99	666.98	3	*2.74E+07*		7.15E+06	0.36%	18	9.62 ± 2.30	
A iso	220-243	DGEDQTQDTELVETRPAGDGTFQK	879.74	879.74	3	*8.02E+07*		4.20E+07	0.21%	22	14.3 ± 2.59	
B40	132-145	SWTAADTAAQISQR (3+)	502.58	502.58	3	*2.61E+07*		4.77E+06	0.34%	10	10.3 ± 2.79	12.1 ± 1.51
B40	132-145	SWTAADTAAQISQR (2+)	753.37	753.37	2	*4.99E+07*		6.05E+06	0.26%	9	11.9 ± 2.90	
B iso	220-239	DGEDQTQDTELVETRPAGDR	744.67	744.67	3	*1.49E+08*		4.51E+07	0.32%	23	14.0 ± 1.99	
Cw1	112-131	GYDQYAYDGKDYIALNEDLR	794.70	794.73	3	*6.15E+07*		4.51E+06	0.55%	18	25.9 ± 7.26	23.7 ± 2.72
C iso	132-145	SWTAADTAAQITQR	760.38	760.38	2	*2.15E+07*		3.08E+06	0.59%	9	25.4 ± 4.98	
C iso	318-341	GGSCSQAASSNSAQGSDESLIACK	791.34	791.34	3	*4.87E+06*		7.93E+05	0.40%	14	15.6 ± 4.71	
C iso	257-273	YTCHVQHEGLPEPLTLR (3+)	684.01	684.01	3	*8.83E+06*		2.78E+06	0.84%	10	34.8 ± 5.85	
C iso	257-273	YTCHVQHEGLPEPLTLR (4+)	513.26	513.26	4	*4.50E+06*		6.47E+06	0.35%	9	20.7 ± 6.35	

**HD6** [A1, 24; B8, 27; Cw2, Cw7] [a]												
						**Unlabeled**		**72 h labeled** (p = 5.11%) [e]				
**Molecule**	**Residues**	**Sequence** [b]	***m/z* (pred)** [c]	***m/z* (obs)**	***z***	**Abundance** [g]	**RMSD (%)** [d]	**Abundance**	**RMSD (%)**	**n**	***k* (%/h)** [f]	**k_{iso} (%/h)**
A1	115-131	QDAYDGKDYIALNEDLR	666.99	666.99	3	*7.36E+07*		4.98E+07	0.34%	20	7.23 ± 0.29	10.7 ± 1.26
A24	36-44	FDSDAASQR	498.72	498.72	2	*5.96E+07*		1.32E+07	0.39%	10	10.9 ± 4.33	
A24	49-66	APWIEQEGPEYWDEETGK	1082.48	1082.479	2	*2.33E+07*		1.42E+07	0.55%	21	14.4 ± 2.97	
A24	316-341	KGGSYSQAASSDSAQGSDVSLTACKV	854.40	854.40	3	*8.91E+06*		3.60E+07	0.60%	17	8.14 ± 2.98	
A iso	22-35	FIAVGYVDDTQFVR	815.42	815.42	2	*1.91E+08*		1.94E+08	0.23%	9	13.6 ± 4.12	
A iso	220-243	DGEDQTQDTELVETRPAGDGTFQK	879.74	879.74	3	*2.08E+08*		2.49E+08	0.42%	26	12.6 ± 2.49	
B8	7-17	YFDTAM[ox]SRPGR	658.81	658.81	2	*9.63E+06*		4.20E+06	0.38%	12	9.79 ± 2.30	13.1 ± 1.26
B8	171-181	YLENGKDTLER	669.34	669.34	2	*4.81E+07*		6.29E+06	0.71%	9	20.2 ± 5.03	
B8	112-131	GHNQYAYDGKDYIALNEDLR	785.71	785.71	3	*3.67E+07*		3.92E+07	0.38%	16	10.7 ± 2.06	
B27	22-35	FITVGYVDDTLFVR	822.94	822.94	2	*1.72E+08*		1.22E+08	0.43%	8	18.3 ± 6.48	
B iso	49-62	APWIEQEGPEYWDR	592.61	592.61	3	*7.65E+06*		7.26E+06	0.88%	15	9.46 ± 1.70	
B iso	220-239	DGEDQTQDTELVETRPAGDR	744.67	744.68	3	*3.29E+08*		2.40E+08	0.23%	25	14.1 ± 2.98	

**HD7** [A24, 30; B13, 27; Cw2, w6] [a]												
						**Unlabeled**		**72 h labeled** (p = 4.76%) [e]				

Molecule	Residues	Sequence [b]	*m/z* (pred) [c]	*m/z* (obs)	*z*	Abundance [g]	RMSD (%) [d]	Abundance	RMSD (%)	n	*k* (%/h) [f]	k_{iso} (%/h)
A iso	36-44	FDSDAASQR	498.72	498.73	2	*6.29E+07*		2.62E+06	0.59%	8	12.4 ± 3.02	14.4 ± 1.93
A iso	220-243	DGEDQTQDTELVETRPAGDGTFQK	879.74	879.75	3	*5.00E+07*		1.70E+08	0.46%	23	16.4 ± 2.86	
B13	22-35	FITVGYVDDTQFVR	830.42	830.43	2	*7.61E+07*		1.28E+08	0.24%	7	11.9 ± 1.22	11.5 ± 1.32
B27	22-35	FITVGYVDDTLFVR	822.94	822.95	2	*5.64E+07*		6.79E+07	0.36%	7	12.4 ± 0.60	
B iso	49-62	APWIEQEGPEYWDR	888.41	888.42	2	*9.30E+07*		2.66E+07	0.29%	13	13.3 ± 3.55	
B iso	220-239	DGEDQTQDTELVETRPAGDR	1116.50	1116.52	2	*2.92E+07*		9.29E+06	0.57%	22	7.99 ± 2.69	

**HD8** [A2, A2, B15, B35, C10 *(*0302)* , C4] [a]												
						**Unlabeled**		**72 h labeled** (p = 4.96%) [e]				
Molecule	Residues	Sequence [b]	*m/z* (pred) [c]	*m/z* (obs)	*z*	Abundance	RMSD (%) [d]	Abundance	RMSD (%)	n	*k* (%/h) [f]	k_{iso} (%/h)
A2	317-340	GGSYSQAASSDSAQGSDVSLTACK	1167.51	1167.52	2	2.46E+06	1.61%	4.37E+06	0.28%	14	9.00 ± 6.29	11.0 ± 1.80
A2	220-243	DGEDQTQDTELVETRPAGDGTFQK	879.74	879.74	3	1.40E+08	0.06%	1.16E+09	0.63%	21	12.7 ± 2.82	
B15	152-157	EAEQWR	409.69	409.70	2	1.27E+07	1.82%	2.88E+08	0.50%	7	11.7 ± 4.02	14.3 ± 1.46
B iso	49-62	APWIEQEGPEYWDR	888.41	888.41	2	1.98E+08	0.17%	3.25E+09	0.56%	12	14.3 ± 2.14	
B iso	317-338	GGSYSQAASSDSAQGSDVSLTA	1023.45	1023.46	2	4.59E+07	4.20%	5.75E+08	0.55%	13	18.2 ± 1.30	
B iso	220-239	DGEDQTQDTELVETRPAGDR	744.67	744.68	3	2.51E+08	0.16%	6.03E+09	0.83%	19	14.2 ± 2.61	
C10	18-35	GEPHFIAVGYVDDTQFVR	684.01	684.01	3	1.40E+07	1.39%	1.62E+08	0.14%	13	14.9 ± 5.31	15.9 ± 2.52
C iso	122-131	DYIALNEDLR	611.31	611.31	2	4.31E+06	2.11%	3.06E+07	0.31%	12	15.6 ± 5.43	
Ciso	132-145	SWTAADTAAQITQR	760.38	760.38	2	1.44E+07	0.31%	1.72E+08	0.17%	13	15.2 ± 1.56	
Ciso	256-273	YTCHVQHEGLPEPLTLR	684.01	684.01	3	5.19E+06	1.41%	8.48E+07	0.40%	12	18.8 ± 12.3	

**HD8** [A2, A2, B15, B35, C10 *(*0302)* , C4] [a] **(LPS stimulated)**												
						**Unlabeled**		**72 h labeled** (p = 4.65%) [e]				
Molecule	Residues	Sequence [b]	*m/z* (pred) [c]	*m/z* (obs)	*z*	Abundance	RMSD (%) [d]	Abundance	RMSD (%)	n	*k* (%/h) [f]	k_{iso} (%/h)
A2	49-65	APWIEQEGPEYWDGETR	1031.96	1031.97	2	1.92E+08	0.67%	6.55E+07	0.81%	9	6.87 ± 0.65	(5.18 ± 0.88)
A2	220-243	DGEDQTQDTELVETRPAGDGTFQK	879.74	879.74	3	8.20E+08	0.11%	5.61E+07	0.50%	28	3.58 ± 0.60	(p = 0.031)
B15	152-157	EAEQWR	409.69	409.70	2	1.62E+08	0.75%	6.77E+06	0.39%	8	4.15 ± 0.69	4.49 ± 0.90
B35	98-108	MYGCDLGPDGR	620.76	620.76	2	5.16E+07	2.11%	1.01E+07	0.86%	6	4.54 ± 4.43	
B iso	49-62	APWIEQEGPEYWDR	888.41	888.41	2	1.58E+09	0.08%	1.43E+08	0.41%	15	4.65 ± 0.83	
B iso	220-239	DGEDQTQDTELVETRPAGDR	744.67	744.68	3	2.05E+09	0.18%	1.13E+08	0.78%	24	4.64 ± 0.75	
C10	18-35	GEPHFIAVGYVDDTQFVR	684.01	684.01	3	9.59E+07	1.54%	9.69E+06	1.00%	12	8.54 ± 1.09	9.06 ± 0.72
C iso	122-131	DYIALNEDLR	611.31	611.31	2	5.40E+07	1.05%	4.33E+06	0.88%	13	10.1 ± 1.65	
Ciso	132-145	SWTAADTAAQITQR	760.38	760.38	2	1.13E+08	0.48%	9.41E+06	0.16%	15	8.58 ± 1.48	

Notes:

[a] HLA types: given as serotypes (numerically equivalent to 2-digit genotypes except where noted in parentheses, in *italics).*
By default, the 4-digit subtypes most prevalent in Caucasians were used for alignments (usually *xx:01). B27 was assumed to be B*27:05.

[b] All peptides used for kinetic analysis are shown (single-letter code). Residue numbering is based on the mature protein sequence, with signal sequence cleavage as per the IMGT/HLA database.

[c] m/z: mass to charge ratio, predicted from monoisotopic elemental masses or observed in Orbitrap mass spectra as indicated. Abundances were summed over all mass isotopomers used in the analysis.

[d] RMSD, root mean square deviation of observed mass isotopomer distributions, as compared with MIDA models.

[e] "p" represents measured 2H2O enrichment in media. "n", number of labeling sites that yields best fit to MIDA models at this enrichment.

[f] "*k*" represents fractional synthesis rates (% per hour, mean ± SE) based on single-exponential curve fit to data. "k_{iso}" represents pooled data for isotypes shown in Figs 5-8.
Where k_{iso} values are shown in parentheses, the *k* values for individual peptides were significantly different (p value shown, F test).

[g] Abundances in *Italics* / RMSD blank: some unlabeled sample data acquired at 30,000 resolution, resulting in bias (R.B. et al., unpublished data).
Where abundances are in plain font reported and RMSD values are given, mass spectra were acquired at 7,500 resolution.
For consistency, fractional synthesis rates for all MoDC samples were calculated using the unlabeled MIDA model for baseline subtraction.
(Where available, baseline subtraction using unlabeled data acquired at 7,500 resolution yielded similar results.)
